# Treatment of Idiopathic Membranous Nephropathy for Moderate or Severe Proteinuria: A Systematic Review and Network Meta-Analysis

**DOI:** 10.1155/2022/4996239

**Published:** 2022-04-23

**Authors:** Miaomiao Chen, Jiarong Liu, Yi Xiong, Gaosi Xu

**Affiliations:** ^1^Department of Nephrology, The Second Affiliated Hospital of Nanchang University, Nanchang, Jiangxi, China; ^2^Grade 2018, The First Clinical Medical College of Nanchang University, Nanchang, Jiangxi, China

## Abstract

**Objective:**

Numerous studies have demonstrated that the efficacy of drugs differs in idiopathic membranous nephropathy (IMN) patients with moderate or high proteinuria. However, there is no systematic comparison confirming it. This network meta-analysis (NMA) was performed to respectively compare the efficacy of ten IMN treatments in patients with moderate and high proteinuria and compare the risk of adverse events with 10 IMN regimens.

**Methods:**

Randomized controlled trials (RCTs) and observational studies analyzing the main therapeutic regimens for IMN were included from some databases. Network comparisons were performed to analyze the rates of total remission (TR), bone marrow suppression, and gastrointestinal symptoms. The surface under the cumulative ranking area (SUCRA) was calculated to rank interventions.

**Results:**

Seventeen RCTs and eight observational studies involving 1778 patients were pooled for comparison of ten interventions. Steroid + tacrolimus (TAC) showed the highest probabilities of TR whether patients had severe proteinuria or not (SUCRA 89.5% and 88.9%, separately). Rituximab (RTX) was more beneficial for TR on patients with proteinuria <8 g/d (SUCRA 66.0%) and was associated with a lower risk of bone marrow suppression and gastrointestinal symptoms (SUCRA 21.7% and 21.4%, separately). TAC + RTX and steroids + cyclophosphamide induced the highest rates of bone marrow suppression (SUCRA 90.6% and 88.3%, separately) and gastrointestinal symptoms (SUCRA 86.0% and 72.1%, separately).

**Conclusions:**

Steroids + TAC showed significant efficacy in patients with all degrees of proteinuria, while RTX was more effective in patients with moderate proteinuria and was safer in bone marrow suppression and gastrointestinal symptoms.

## 1. Introduction

Idiopathic membranous nephropathy (IMN) is one of the most common causes of nephrotic syndrome in adults [1]. It is an immune-mediated disease with subepithelial immune complex deposition and glomerular basement membrane changes [2]. In 2009, Beck et al. published a seminal paper reporting that nearly 70% of adult IMN patients had IgG4 antibodies to podocyte-expressed M-type phospholipase A2 receptor (PLA2R) [2–4], whereas the thrombospondin type-1 domain-containing 7A was the target in one to five percent of patients [5]. These findings correlate significantly with clinical disease activity and can be used to monitor disease activity [4]. IMN is characterized by spontaneous remission and recurrence. About 40% of people experience spontaneous remissions and 15% to 30% have relapses. 50% of patients went on to develop the nephrotic syndrome, of which 30% progressed to end-stage renal disease [6].

The variable natural course of IMN makes its treatment controversial. Many patients are advised to receive conservative treatment and continue immunosuppressive therapy after 6–12 months if partial or complete remission is not achieved [7, 8]. The Kidney Disease: Improving Global Outcomes (KDIGO) 2021 guidelines [7] recommend rituximab (RTX) therapy, cyclophosphamide (CYC) combined glucocorticoids therapy for 6 months, or tacrolimus (TAC) based therapy at least 6 months. And treatments need to be selected according to the patient's risk assessment. However, there is still no high-level meta-analysis providing reliable therapeutic strategies for IMN patients with different risk levels. Although immunosuppressive therapy has been used for many years, it remains difficult for clinicians to choose between benefit and safety because of the serious adverse events [9].

Infection, bone marrow suppression, and gastrointestinal symptoms are common side effects along with immunosuppressive therapy. Only one traditional pairwise meta-analysis compared the risk of steroids + CYC and TAC on gastrointestinal symptoms [10]. No network meta-analysis reported the risk of steroids combined with immunosuppressive agents in bone marrow suppression and gastrointestinal symptoms. Considering the direct comparative evidence is lacking, and the traditional pairwise meta-analyses are not enough to synthesize all the evidence and rank treatments simultaneously, it is necessary to perform this network meta-analysis (NMA) to remedy the deficiencies.

In this study, we conducted an NMA to explore the efficacy of ten treatments for IMN patients with moderate or severe proteinuria. The risks of bone marrow suppression and gastrointestinal symptoms associated of different treatment regimens for IMN were also compared.

## 2. Methods

### 2.1. Data Sources and Search Strategy

This network meta-analysis was performed according to PRISMA (Preferred Reporting Items for Systematic Reviews and Meta-Analyses) guidelines [11]. From inception until August 1, 2021, a pre-established retrieval strategy was used to screen eligible studies about the IMN treatments from PubMed, Web of Science, Embase, Cochrane Library, and Medline five databases. The search strategy was as follows: [(Membranous Glomerulonephritides) OR (Membranous Glomerulonephritis) OR (Membranous Glomerulopathy) OR (Membranous Nephropathy) OR (Extramembranous Glomerulopathy) OR (Membranous Glomerulonephropath) OR (Idiopathic Membranous Glomerulonephritis) OR (Idiopathic Membranous Glomerulonephritides) OR (Idiopathic Membranous Nephropathy)] AND [(Tacrolimus) OR (Cyclophosphamide) OR (Rituximab) OR (Cyclosporin) OR (Mycophenolate mofetil) OR (Steroids)]. Studies included in relevant publication references were also reviewed to avoid omissions. In addition, no language restrictions were set in the search process.

### 2.2. Selection Criteria

The selection criteria for including publications were as follows: (1) the study type should be randomized controlled trials (RCTs), cohort control studies, or case-control studies (Supplement 1 for specific selection criteria); (2) patients in the studies must have biopsy-proven IMN and nephrotic range proteinuria (urinary protein excretion >3.5 g/d); (3) each study should report the number of patients with total remission (TR) or various adverse events. TR was defined as either complete or partial remission, complete and partial remission were respectively defined as proteinuria of <0.3 g/day and <3.5 g/day, with at least a 50% reduction in baseline values and stable renal function; and (4) interventions for studies should include TAC, CYC, cyclosporine A (CsA), RTX, steroids, nonimmunosuppressive antiproteinuric treatment (NIAT), steroids + TAC, steroids + CYC, steroids + CsA, steroids + mycophenolate mofetil (MMF), and TAC + RTX. Publications conforming to the following criteria were excluded: (1) study participants were not adults (younger than 16 years); (2) the study was not designed to compare the efficacy and safety of different medications for IMN; (3) the treatment had not to be first-line or patients had received immunotherapies before the study; (4) patients with secondary membranous nephropathy, IMN after kidney transplantation, or atypical membranous nephropathy; (5) drugs included in the publication were not involved in our study, or publications compared the same drug in terms of administration route or dosage, etc.

### 2.3. Data Extraction and Quality Evaluation

After screening the title and abstract, the article meeting the inclusion criteria was obtained for evaluation and data extraction. Information gleaning from the pooled studies included the first author's name, publication year, country, type of intervention, and participant characteristics. All included studies were assessed based on quality. For the RCTs, we evaluated the risk of bias (selection bias, performance bias, detection bias, attrition bias, reporting bias, and other bias) using Review Manager 5.4 software [12–14]. Each of these six domains could be classified as “low risk of bias,” “unclear risk of bias,” or “high risk of bias” [14]. For cohort studies and case-control studies, the Newcastle-Ottawa scale, which adopted the semi-quantization principle of the star system, was used to evaluate the quality of selection, comparability, and exposure/outcome [15]. Two authors (MMC and YX) independently extracted the data and assessed the quality of the studies, and differences were resolved through the third reviewer (GSX).

### 2.4. Statistical Analysis

The number of patients experiencing TR, bone marrow suppression, or gastrointestinal symptoms were extracted from publications. Odds ratios (ORs) and corresponding 95% confidence intervals (CIs) were used to compare the efficacy and safety of different drugs. STATA 14.0 software was used to conduct the conventional pairwise meta-analysis for determining the effects of different IMN agents. In this NMA, we employed STATA 14.0 (“mvmeta” and “network” packages) to draw the trial network plots and assess for publication bias and R 4.1.1 (“ggplot2,” “JAGS” and “gemtc” packages) to conduct statistical analysis. R 4.1.1 was employed for a Bayesian frame structure, while STATA 14.0 was used in a frequentist framework. We generated 1,000,000 simulations for each of the two sets of different initial values and discarded the first 50,000 simulations as the burn-in period. Then the convergence and density diagrams were examined by using Brooks-Gelman-Rubin diagnostics and trace plots. The surface under the cumulative ranking curve (SUCRA), simply transforming the mean rank, was used to provide a hierarchy of the treatments [3]. Higher SUCRA values manifested higher treatment grades. The node-splitting method was used to compare inconsistencies between direct and indirect evidence. This method split the same comparison into direct and indirect comparisons and used *p* values to assess the difference between them [18]. Pairwise and network heterogeneity were evaluated using *I*^2^, and *I*^2^ more than 50% indicated significant heterogeneity. Meta-regression was conducted to investigate the source of heterogeneity by R software.

## 3. Results

### 3.1. Baseline Characteristics of Enrolled Studies

**Figure 1:**
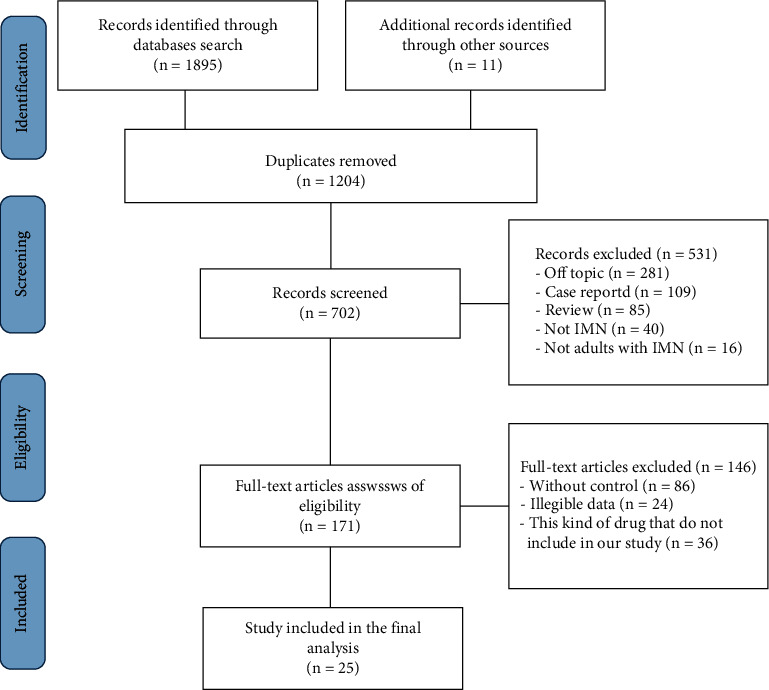
Flowchart for selection of articles to be included in the NMA.

The flow of the systematic review is shown in Figure 1. In 1906, potentially relevant articles were screened, and 1204 duplicate studies were deleted. Only 25 [4, 19–42] publications involving 1778 patients were included in the final analysis. Of the 25 studies, seventeen RCTs, four prospective studies, three retrospective studies, and one case-control study were included. The quality of the included studies ranged from high to medium (Supplement 2). The baseline characteristics of the included studies are summarized in Table 1. It can be seen that the patients from 11 articles had pre-study proteinuria greater than 8 g/d (severe proteinuria), while the patients of the other 14 studies had pre-study proteinuria of less than 8 g/d (moderate proteinuria). Bone marrow suppression and gastrointestinal symptoms were reported in ten and thirteen studies, respectively. There were ten IMN interventions included in the study, namely, steroids + CYC, steroids + TAC, steroids + CsA, CsA, TAC, TAC + RTX, NIAT, RTX, steroids, and steroids + MMF.

### 3.2. Network Structure, Consistency, and Heterogeneity

**Figure 2:**
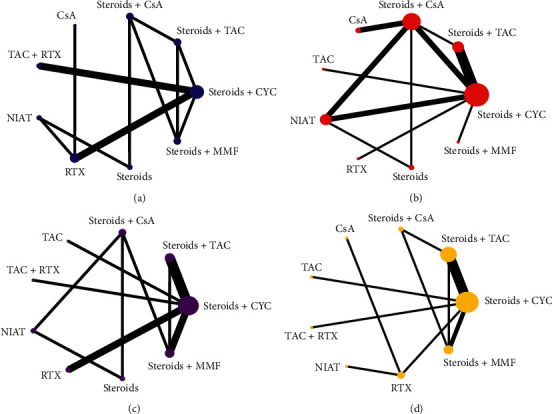
Network plots of the comparisons between different therapies: (a) total remission (prestudy proteinuria >8 g/d); (b) total remission (prestudy proteinuria <8 g/d); (c) bone marrow suppression; (d) gastrointestinal symptoms. RTX, rituximab; CsA, cyclosporin A; CYC, cyclophosphamide; TAC, tacrolimus; NIAT, nonimmunosuppressive antiproteinuric treatment; MMF, mycophenolate mofetil.

Figure 2 shows a network plot of treatment comparisons. The number of interventions was nine for TR (prestudy proteinuria >8 g/d), TR (prestudy proteinuria <8 g/d), bone marrow suppression, and gastrointestinal symptoms. The size of the nodes correlated with the intervention's sample size. And the straight line whose thickness corresponded to the test number of direct comparison was used to connect different treatment regimens. As shown in Figure 2, the sample size and comparison times of different interventions were different.

The diagnostic and trace plots demonstrated that the convergence of this NMA was satisfactory. As presented in Supplement 3, the node splitting methods were used to conduct consistency analysis, and all the *p* values were greater than 0.05, which indicated that our work had high consistency and reliability. In the heterogeneity analysis (Supplement 4), significant heterogeneity could be found in the comparison of TAC + RTX and steroids + CYC for TR (prestudy proteinuria >8 g/d), steroids + CsA and NIAT for TR (prestudy proteinuria <8 g/d), and steroids + MMF and steroids + CYC for bone marrow suppression and gastrointestinal symptoms. That was why we chose the random-effects model and did meta-regression to look for the sources of heterogeneity.

### 3.3. Pairwise Meta-Analysis

The results of the pairwise meta-analysis are shown in supplement 5.

### 3.4. Network Meta-Analysis

#### 3.4.1. TR (Prestudy Proteinuria >8 g/d)

**Figure 3:**
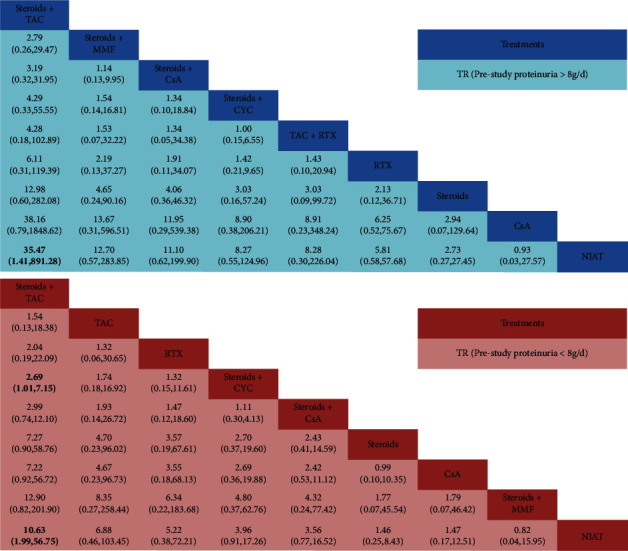
Summary of results of the network meta-analysis (NMA). For each comparison, the random effects model odd ratios (ORs) and 95% confidence intervals are provided. The results of the plots are read from top to bottom and left to right. An OR >1 indicates that the treatment in the top left is more effective than the comparator treatment. Significant results are shown in bold. RTX, rituximab; CsA, cyclosporin A; CYC, cyclophosphamide; TAC, tacrolimus; NIAT, nonimmunosuppressive antiproteinuric treatment; MMF, mycophenolate mofetil.

TR (prestudy proteinuria >8 g/d) was reported in 11 publications involving 875 patients. Nine regimens including five immunosuppressive drugs were included: steroids + CYC (5 trials, 212 patients), steroids + TAC (2, 45), steroids + CsA (3, 64), CsA (1, 63), TAC + RTX (2, 90), NIAT (2, 76), RTX (4, 214), steroids (2, 62), and steroids + MMF (2, 49). As illustrated in Figure 3, compared with NIAT, steroids + TAC had significant advantages inducing TR (OR = 35.47, 95% CI: 1.41∼891.28) on patients with proteinuria >8 g/d. The results of other treatment regimens were not statistically significant.

**Figure 4:**
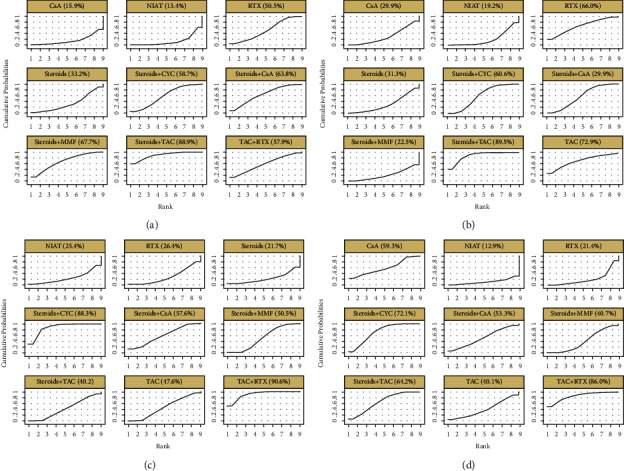
Rankings of SUCRA for all treatments. (a) Total remission (prestudy proteinuria >8 g/d); (b) total remission (prestudy proteinuria <8 g/d); (c) bone marrow suppression; (d) gastrointestinal symptoms. RTX, rituximab; CsA, cyclosporin A; CYC, cyclophosphamide; TAC, tacrolimus; NIAT, nonimmunosuppressive antiproteinuric treatment; MMF, mycophenolate mofetil.

The SUCRA of TR (prestudy proteinuria >8 g/d) for treatments is shown in Figure 4. Steroids + TAC and steroids + MMF were ranked the first and second (SUCRA of 88.9% and 67.7%, respectively), followed by steroids + CsA and steroids + CYC (SUCRA of 63.8% and 58.7%, respectively). NIAT was ranked as the worst treatment (SUCRA of 13.4%).

#### 3.4.2. TR (Prestudy Proteinuria <8 g/d)

14 studies and 903 patients were pooled in the NMA of TR (prestudy proteinuria <8 g/d). The therapeutic schedules involved were as follows: steroids + CYC (10 trials, 271 patients), steroids + TAC (5, 176), steroids + CsA (6, 162), CsA (2, 45), TAC (1, 27), NIAT (3, 113), RTX (1, 26), steroids (1, 72), and steroids + MMF (1, 11). The staircase diagram (Figure 3(b)) showed that steroids + TAC was a more effective treatment and had a significantly higher rate of TR than steroids + CYC and NIAT (OR = 2.69, 95% CI: 1.01∼7.65; OR = 10.63, 95% CI: 1.99∼56.75) in patients with proteinuria <8 g/d.

Like the results of NMA on TR (prestudy proteinuria >8 g/d), steroids + TAC still had the highest rate of TR (SUCRA of 89.5%) in IMN patients with lower proteinuria (<8 g/d). It was followed by TAC, RTX, and steroids + CYC (SUCRA of 72.9%, 66.0%, and 60.6%, respectively). NIAT was the lowest in achieving TR (Figure 4(b)).

#### 3.4.3. Adverse Events

The incidences of adverse events for the 10 therapeutic regimens are shown in Supplement 6. Infection, bone marrow suppression, and gastrointestinal symptoms were the most common adverse events, which was the same as what Zheng et al. concluded [41]. Because the incidence of infection had been well documented in an NMA [42], we were mainly concerned about the risk of bone marrow suppression and gastrointestinal symptoms in IMN treatments.

**Figure 5:**
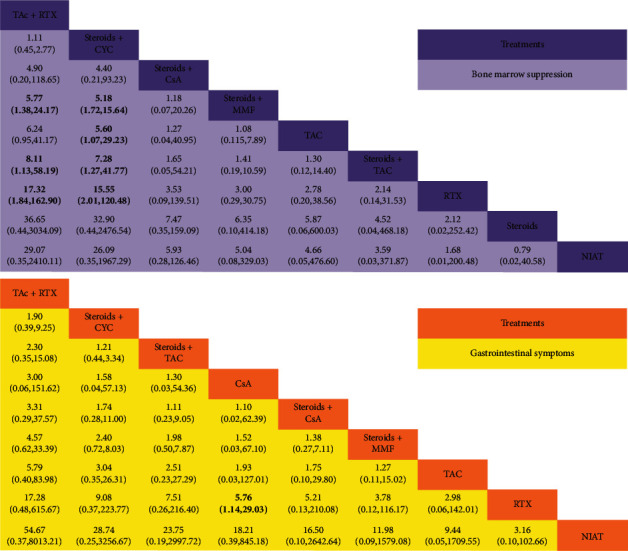
Summary of the results of the network meta-analysis (NMA). For each comparison, the random effects model odd ratios (ORs) and 95% confidence intervals are provided. The results of the plots are read from top to bottom and left to right. An OR >1 indicates that the treatment in the top left is worse/less safe than the comparator treatment. Significant results are shown in bold. RTX, rituximab; CsA, cyclosporin A; CYC, cyclophosphamide; TAC, tacrolimus; NIAT, nonimmunosuppressive antiproteinuric treatment; MMF, mycophenolate mofetil.

There were ten trials consisting of 919 patients, and nine treatments reported the incidence of bone marrow suppression. The regimens contained the following: steroids + CYC (8 trials, 336 patients), steroids + TAC (3, 93), steroids + CsA (2, 68), TAC (1, 30), TAC + RTX (1, 43), NIAT (1, 57), RTX (2, 137), steroids (1, 72), and steroids + MMF (3, 83). As shown in Figure 5, TAC + RTX had a higher risk of bone marrow suppression compared to RTX, steroids + TAC, and steroids + MMF (OR = 17.32, 95% CI: 1.84∼162.9; OR = 8.11, 95% CI: 1.13∼58.19; and OR = 5.77, 95% CI: 1.38∼24.17, respectively). RTX, steroids + TAC, TAC, and steroids + CsA, compared with steroids + CYC, were associated with a lower rate. TAC + RTX and steroids + CYC were most likely to be ranked first and second in terms of bone marrow suppression (SUCRA of 90.6% and 88.3%, respectively), whereas steroids, NIAT, and RTX seemed to rank lower (SUCRA of 21.7%, 25.4%, and 26.4%, respectively).

Thirteen studies were enrolled to calculate the incidence of gastrointestinal symptoms. The analysis included 1,075 patients with nine treatments. Regimens were listed as follows: steroids + CYC (8 trials, 385 patients), steroids + TAC (6, 196), steroids + CsA (2, 33), CsA (1, 65), TAC (1, 30), TAC + RTX (1, 43), NIAT (7, 38), RTX (3, 202), and steroids + MMF (3, 83). Figure 5 shows a comparison of all treatments for gastrointestinal symptoms. Only CsA was associated with a higher rate (OR = 5.76, 95% CI: 1.14∼29.3) in the cause of gastrointestinal symptoms. As illustrated in Figure 4(d), TAC + RTX and steroids + CYC were ranked the worst/unsafe or second-worst (SUCRA of 86.0% and 72.1%, respectively) regarding gastrointestinal symptoms. On the contrary, NIAT and RTX had the lowest ranks (SUCRA of 12.9% and 21.4%, respectively) compared to the other included regimens.

#### 3.4.4. Meta-Regression and Publication Bias

A metaregression was performed to explore the heterogeneity source. As covariates, patients' age and study duration were adjusted for TR, bone marrow suppression, and gastrointestinal symptoms. The results showed that patients' age and study duration were associated with the heterogeneity of TR and bone marrow suppression, while there was no significant effect on gastrointestinal symptoms (Supplement 7). The comparison-adjusted funnel plots were also made, and no significant publication bias was detected (Supplement 8).

## 4. Discussion

In this study, we performed an NMA to assess the efficacy and safety of different treatments for IMN. Twenty-five articles including 1778 patients were included in our study. To explore the effectiveness of drugs on people with proteinuria of more than or less than 8 g/d, the incidence of TR was divided into two groups. As illustrated in our results, steroid + TAC was considered the most efficient therapy for TR regardless of patients' proteinuria levels. For patients with prestudy proteinuria >8 g/d, the treatment of steroid + MMF and steroid + CYC had a similar beneficial effect for total remissions of proteinuria, followed by steroid + CYC, TAC + RTX, and RTX. However, for patients with proteinuria <8 g/d, TAC, steroid + CYC, and RTX seemed to be better treatments than others in achieving TR. Then, we compared the risk of bone marrow suppression and gastrointestinal symptoms with different treatments. The present NMA demonstrated that TAC + RTX and steroid + CYC were associated with higher risks of bone marrow suppression and gastrointestinal reactions, namely the higher ranks of SUCRA. Furthermore, RTX has been demonstrated to induce a low risk of bone marrow suppression and gastrointestinal reactions, which was similar to the risk of NIAT.

Calcineurin inhibitors (CsA or TAC), as monotherapy or in combination with low-dose glucocorticoids, can improve treatment outcomes and reduce toxicity [43, 44]. A traditional meta-analysis recently reported that steroids + TAC could get a high value of TR when compared with CYC on a 6-month treatment in IMN [45]. Our research came to a similar conclusion, and we further confirmed that steroids + TAC were the most effective in patients with proteinuria greater than 8 g/d and less than 8 g/d. Meanwhile, the KDIGO 2021 guidelines recommend the use of steroids + TAC for both medium-risk (proteinuria less than 8 g/d but greater than 3.5 g/d) and high-risk patients (proteinuria more than 8 g/d), which also confirms our conclusion. Most studies reported both that steroids + TAC and steroids + CsA were effective for adult IMN [39, 44, 46, 47]. Our study came to the same conclusion. Although the beneficial effects of steroids + TAC compared with steroids + CsA did not reach statistical significance, it was probably that steroids + TAC had superiority in terms of rank.

Alkylating agents (chlorambucil and CYC) are immunosuppressants that have been proven to be effective in preventing end-stage renal disease and death. Although the efficacy between chlorambucil and CYC was comparable, CYC was safer and more widely used by clinicians [48, 49], which was the reason why we did not include chlorambucil. The present NMA demonstrated there was no statistical difference in the efficacy between steroids + CYC and steroids + TAC for patients with proteinuria levels >8 g/d; however, steroids + TAC appeared to be more effective in patients with proteinuria <8 g/d. As far as we know, this was a new conclusion. Some articles had reported that steroids + TAC may be more therapeutic than steroids + CYC in TR; however, they did not evaluate the efficacy of the drug based on the patients' risk of kidney function loss. The STARMEN trial indicated that alternating treatment with steroids and CYC was superior to sequential treatment with TAC and RTX in IMN [25], and our conclusions also supported their viewpoints; namely, steroid + CYC had a higher SUCRA than TAC + RTX in TR. Many traditional meta-analyses have reported the good efficacy of RTX in the treatment of IMN [50–52]. Nevertheless, they did not distinguish the effects of RTX on patients with different proteinuria. Our study combining direct and indirect evidence verified that TR was more achievable in patients with proteinuria <8 g/d. The reason may be that severe glomerular damage causes loss of RTX from the urine, reducing the concentration of RTX in the body, which makes treatment less effective in patients with proteinuria >8 g/d. Simultaneously, compared with KDIGO 2012 guidelines [8], KDIGO 2021 guidelines [7] emphasize the role of RTX in patients with a moderate and high risk of IMN, which confirms our conclusion again.

The data regarding other agents were limited. We found steroids alone had little effect in achieving remission of proteinuria, which explains why regimens of steroids combined with immunosuppressants were often used. Steroids + MMF were consistent with good therapeutic effects in getting TR. Since only three articles about steroids + MMF were included, the result of steroids + MMF in the NMA needs to be consolidated by more RCTs.

As far as we are concerned, this is the first study to compare the risks of the main IMN therapeutic regimens in bone marrow suppression and gastrointestinal disease. A network published in 2019 just provided data about the frequency of adverse effects and lacked statistical comparison [41]. Our article compensated for this deficiency by comparing the risk of main IMN treatments in two kinds of adverse events. As mentioned above, TAC + RTX and steroids + CYC were concerned with a high risk of bone marrow suppression and gastrointestinal diseases, which was in sharp contrast to RTX.

As mentioned above, great heterogeneity was found during NMA, which prompted us to search for the possible sources of heterogeneity through meta-regression. Besides, our study also had some limitations. Firstly, the duration of follow-up varied among the included studies, and some were too short. Secondly, three retrospective studies and one case-control study were included, which might have caused significant heterogeneity. Thirdly, the sample size of some studies was small, which reduced the level of evidence in the article. Finally, we failed to register for the review protocol, which was likely to increase reporting bias.

## 5. Conclusions

This was the first NMA comparing the efficacy of ten IMN treatments in patients with different proteinuria, and also the first one reported the risks of IMN treatments in bone marrow suppression and gastrointestinal symptoms. Our study confirmed that steroid + TAC was the most efficient therapy for TR regardless of patients' proteinuria levels. RTX was more effective in achieving TR for patients with proteinuria <8 g/d. Besides, TAC + RTX and steroid + CYC had a similarly high risk of bone marrow suppression and gastrointestinal symptoms, which was significantly different from RTX. For all of this, more RCTs are needed to consolidate our conclusions and investigate the curative efficacy of the implementation period and dosages on drug efficacy.

## Figures and Tables

**Table 1:** Characteristics of the included studies on the comparison of different therapies.

Study	Country	Study design	Study duration (mo)		Interventions	Sample size	Male	Age (y)	Proteinuria baseline
			Treatment	Follow-up					
Coggins [17]	America	RCT	12	36	T: steroids C: NIAT	T: 34 C: 38	T: 22 C: 20	NA	T: 9.4 ± 6 C: 8.3 ± 4
Cattran et al. [18]	America	RCT	6	17	T: steroids C:steroids + CsA	T: 23 C: 28	T: 16 C: 26	T: 49 ± 14 C: 47 ± 11	T: 8.8 ± 4.7 C: 9.7 ± 5.3
Medrano et al. [4]	Spain	Prospective cohort study	6	12	T: steroids + CYC C: TAC + RTX	T: 26 C: 53	T: 18 C: 31	T: 51.8 ± 17.3 C: 51.1 ± 14.2	T: 11.9 ± 4.7 C: 12.3 ± 3.6
Peng et al. [20]	China	Prospective cohort study	6	3	T: steroids + TAC C: steroids + CYC c: steroid + MMF	T: 30 C: 30 C: 30	T: 17 C: 16 c: 14	T: 43.9 ± 13.2 C: 40.8 ± 13.34 c: 39.9 ± 14.3	T: 11.7 ± 3.2 C: 11.9 ± 1.5 c: 12.1 ± 3.7
Dahan et al. [21]	France	RCT	6	18	T: RTX C: NIAT	T: 37 C: 38	T: 28 C: 24	T: 52.6 ± 16.2 C: 55.0 ± 16.2	NA
Li et al. [39]	China	RCT	6	6	T: steroids + TAC C: steroids + CsA	T: 16 C: 15	T: 12 C: 13	T: 39.4 ± 8.8 C: 42.8 ± 8.1	T: 9.5 ± 1.9 C: 9.7 ± 2.5
van den Brand et al. [22]	Netherlands	Retrospective cohort study	6	40	T: RTX C: steroids + CYC	T: 100 C: 103	T: 72 C: 76	T: 51.5 ± 15.9 C: 55.3 ± 12.7	NA
Choi et al. [23]	Korea	RCT	6	12	T:steroids + MMF C:steroids + CsA	T: 21 C: 18	T: 16 C: 9	T: 57.7 ± 10.0 C: 52.7 ± 10.9	T: 8.9 ± 5.9 C: 8.4 ± 3.5
Fervenza et al. [19]	America	RCT	12	24	T: RTX C: CsA	T: 65 C: 65	T: 47 C: 53	T: 51.9 ± 12.6 C: 52.2 ± 12.4	T: 9.4 ± 4.4 C: 9.5 ± 4.7
Fenoglio et al. [24]	Italy	Case-control study	6	24	T: RTX C: RTX c: steroids + CYC	T: 14 C: 14 c: 14	T: 9 C: 9 c: 8	T: 64.4 ± 10.8 C: 61.4 ± 11.5 c: 67.1 ± 17.5	T: 7.5 ± 4.8 C: 5.1 ± 1.4 c: 8.3 ± 4.8
Ferna´ndez-Jua´rez et al. [25]	Italy	RCT	9	18	T: steroids + CYC C: TAC + RTX	T: 43 C: 43	T: 24 C: 31	T: 56.2 ± 12.0 C: 55.2 ± 10.8	T: 7.9 ± 5.0 C: 8.6 ± 3.8
Alexopoulos et al. [26]	Greece	Prospective cohort study	12	26	T: steroids + CsA C: CsA	T: 31 C: 20	T: 19 C: 12	T: 56.0 ± 12.0 C: 61.0 ± 13.0	T: 5.1 ± 2.5 C: 4.9 ± 1.5
Jha et al. [27]	India	RCT	6	120	T: NIAT C: steroids + CYC	T: 53 C: 51	T: 27 C: 30	T: 37.2 ± 12.4 C: 38.0 ± 13.6	T: 5.9 ± 2.2 C: 6.1 ± 2.5
Nayagam et al. [28]	India	Prospective cohort study	6	18	T:steroids + MMF C:steroids + CYC	T: 11 C: 10	T:8 C:7	T:30.2 ± 12.6 C:33.1 ± 12.4	NA
Chen et al. [29]	China	RCT	6	12	T: steroids + TAC C: steroids + CYC	T: 39 C: 34	T: 23 C: 18	T: 47.2 ± 11.9 C: 48.6 ± 11.6	T: 7.7 ± 3.9 C: 7.3 ± 3.9
Kosmadakis et al. [30]	Greece	RCT	9	12	T: steroids + CsA C: steroids + CYC c: NIAT	T: 10 C: 8 c: 10	T: 8 C: 4 c: 5	T: 50.5 ± 4.9 C: 55.4 ± 2.8 c: 51.8 ± 5.4	T: 6.6 ± 1.0 C: 7.0 ± 0.7 c: 5.2 ± 0.8
Shin et al. [31]	Korea	Retrospective cohort study	6	57	T: steroids + CsA C: NIAT c: steroids	T: 50 C: 57 c: 72	T: 25 C: 30 c: 47	T: 52.8 ± 13.9 C: 57.5 ± 11.1 c: 51.36 ± 18.0	T: 7.6 ± 4.1 C: 6.9 ± 3.7 c: 7.9 ± 5.16
He et al. [32]	China	RCT	6	12	T: steroids + CYC C: steroids + TAC	T: 28 C:28	T: 19 C: 20	T: 47.2 ± 13.4 C: 45.4 ± 11.5	T: 6.4 ± 2.2 C: 6.8 ± 2.3
Xu et al. [33]	China	RCT	9	18	T: steroids + CYC C: steroids + TAC	T: 52 C: 48	T: 30 C: 31	T: 57.8 ± 14.8 C: 56.3 ± 13.2	T: 5.1 ± 2.2 C: 5.4 ± 2.5
Li et al. [34]	China	RCT	6	14	T: CsA C: steroids + CsA	T: 14 C: 13	T: 10 C: 10	T: 75.1 ± 8.2 C: 74.8 ± 7.9	T: 7.2 ± 3.4 C: 7.5 ± 3.8
Ramachandran et al. [35]	India	RCT	6	12	T: steroids + TAC C: steroids + CYC	T: 35 C: 35	T: 27 C: 20	T: 38.7 ± 1.9 C: 40.8 ± 10.6	T: 6.8 ± 3.6 C: 5.4 ± 2.7
Omrani et al. [40]	Iran	RCT	6	NA	T: steroids + TAC C: steroids + CsA	T: 34 C: 34	T: 13 C: 16	T: 39.4 ± 13.5 C: 36.2 ± 14.3	T: 3.9 ± 1.1 C: 3.9 ± 1.5
Liang et al. [36]	China	RCT	12	10	T: steroids + CYC C: TAC	T: 28 C: 30	T: 9 C: 16	T: 53.9 ± 10.4 C: 48.2 ± 13.5	T: 6.9 ± 2.2 C: 5.9 ± 2.7
Li et al. [37]	China	Retrospective cohort study	6	6	T: steroids + CYC C: steroids + CsA	T: 23 C: 24	T: 16 C: 13	T: 43.0 ± 12.0 C: 42.0 ± 15.0	T: 7.6 ± 6.1 C: 5.8 ± 3.7
Scolari et al. [38]	Italy	RCT	6	30	T: RTX C: steroids + CYC	T: 37 C: 37	T: 37 C: 37	T: 54 .0 ± 14.0 C: 54.0 ± 17.0	T: 6.7 ± 4.6 C: 6.7 ± 3.1

mo, monthy, years; T, treatment group; C, control group 1; c, control group 2; RTX, rituximab; RCT, randomized controlled trial; CsA, cyclosporin A; CYC, cyclophosphamide; TAC, tacrolimus; NIAT, nonimmunosuppressive antiproteinuric treatment; MMF, mycophenolate mofetil; NA, not available. The data are presented as the mean ± standard deviation.

## Supplementary Materials

Supplementary Materials Supplement 1. The selection criteria with a “PICOS” structure for the enrolled studies. Supplement 2. Risk of bias table for included studies. Supplement 3. Evaluation of inconsistency for outcomes. Supplement 4. Evaluation of heterogeneity analysis. Supplement 5. Results from pairwise meta-analyses. Supplement 6. The occurrence of adverse events. Supplement 7. Evaluation of meta-regression. Supplement 8. Net-funnel of publication bias.
