# Supplementary material for: Treatment of Idiopathic Membranous Nephropathy for Moderate or Severe Proteinuria: A Systematic Review and Network Meta-Analysis
Source: Int J Clin Pract. 2022 Apr 23;2022:4996239. doi: 10.1155/2022/4996239 (PMC9159126; doi:10.1155/2022/4996239)
Supplement: Supplementary Materials — Supplement 1. The selection criteria with a “PICOS” structure for the enrolled studies. Supplement 2. Risk of bias table for included studies. Supplement 3. Evaluation of inconsistency for outcomes. Supplement 4. Evaluation of heterogeneity analysis. Supplement 5. Results from pairwise meta-analyses. Supplement 6. The occurrence of adverse events. Supplement 7. Evaluation of meta-regression. Supplement 8. Net-funnel of publication bias. [file 4996239.f1.zip › 4996239.f1/Supplement 1.docx]

***Supplementary material 1: The selection criteria with a “PICOS” structure for the enrolled studies***

eTable1. The selection criteria with a “PICOS” structure for the enrolled studies:

| Items | Specific Criteria |
| --- | --- |
| Patients | Patients were at least 16 years old and had biopsy-proven idiopathic membranous nephropathy (IMN) |
| Interventions/Comparisons | Interventions/Comparisons included Steroids + CYC, Steroids + TAC, Steroids + CsA, Steroids + MMF TAC + RTX, Steroids , CsA, TAC, RTX, NIAT |
| Outcomes | Outcomes were Total remission (TR), Bone marrow suppression, Gastrointestinal symptoms |
| Study designs | Studies were randomized controlled trials (RCTs), prospective clinical trials, retrospective clinical trials, or case-control studies. |

CYC: cyclophosphamide; TAC, tacrolimus; CsA, [cyclosporin](javascript:;) [A;](javascript:;) MMF, Mycophenolate mofetil; RTX, rituximab; NIAT, nonimmunosuppressive antiproteinuric treatment.
