# Supplementary material for: Treatment of Idiopathic Membranous Nephropathy for Moderate or Severe Proteinuria: A Systematic Review and Network Meta-Analysis
Source: Int J Clin Pract. 2022 Apr 23;2022:4996239. doi: 10.1155/2022/4996239 (PMC9159126; doi:10.1155/2022/4996239)
Supplement: Supplementary Materials — Supplement 1. The selection criteria with a “PICOS” structure for the enrolled studies. Supplement 2. Risk of bias table for included studies. Supplement 3. Evaluation of inconsistency for outcomes. Supplement 4. Evaluation of heterogeneity analysis. Supplement 5. Results from pairwise meta-analyses. Supplement 6. The occurrence of adverse events. Supplement 7. Evaluation of meta-regression. Supplement 8. Net-funnel of publication bias. [file 4996239.f1.zip › 4996239.f1/Supplement 2.docx]

# *Supplementary material 2: Risk of bias table for included studies*

eTable1. Quality evaluation of enrolled RCTs according to Cochrane Handbook

| **Study** | **Random sequence generation** | **Random concealment** | **Blinding of participants and personnel** | **Blinding of outcome assessment** | **Incomplete outcome data** | **Selective reporting** | **Other bias** |
| --- | --- | --- | --- | --- | --- | --- | --- |
| Coggins 1979 | The trial only reported “randomized”. | No information was provided. | No information was provided. | No information was provided. | The trial did not report any dropouts. | Results of all primary outcomes were reported. | Unclear. |
| Cattran 2001 | Randomization was performed by the clinical coordinating center from a table of random numbers. | Randomization was stratified by center in blocks of two to ensure a balance between groups. | The patients were masked in regards to active versus placebo assignment, but the physicians were not for safety reasons and because the end points were objective and measured centrally by a lab blinded to patient designation. | No information was provided. | The trial did not report any dropouts. | Results of all primary outcomes were reported | Unclear |
| Fervenza 2009 | This was an open-label, investigator-initiated trial. | No information was provided. | No information was provided. | No information was provided. | The trial did not report any dropouts. | Results of all primary outcomes were reported. | Unclear. |
| Dahan 2016 | The trial only reports “randomized”. | No information was provided. | The trial was not blinded. | Data were collected in a paper case report form and entered into a database. Data assessors were blinded to treatment allocation. | The trial did not report any dropouts. | Results of all primary outcomes were reported. | Unclear. |
| Li Q 2017 | Patients were randomized into two groups according to a randomization list generated from the table of random numbers. | No information was provided. | No information was provided. | No information was provided. | The trial did not report any dropouts. | Results of all primary outcomes were reported. | Unclear. |
| Choi 2018 | The table of random numbers was generated using the SAS randomization program. | Allocation concealment was done by sealed sequentially numbered opaque envelopes. They were consecutively numbered and bottles were provided to the patients according to the number allocated | No information was provided. | No information was provided. | The trial did not report any dropouts. | Results of all primary outcomes were reported. | Unclear. |
| Ferna´ndez-Jua´rez 2021 | The trial used a random number–producing algorithm in central computer systems for simple randomization. | The subject numbers were assigned sequentially as each subject entered the study. | No information was provided. | No information was provided. | The trial did not report any dropouts. | Results of all primary outcomes were reported. | Unclear. |
| Jha 2007 | This was an open-label, investigator-initiated trial. | No information was provided. | No information was provided. | No information was provided. | The trial did not report any dropouts. | Results of all primary outcomes were reported. | Unclear. |
| Chen M 2010 | Randomization was performed by a clinical coordinating center  using a table of random numbers and was stratified by centers. | Allocation concealment was performed by enclosing assignments  in sequentially numbered, opaque-closed envelopes. | No information was provided. | No information was provided. | The trial did not report any dropouts. | Results of all primary outcomes were reported. | Unclear. |
| Kosmadakis 2010 | The trial only reported “randomized” | The person doing the  randomization was blinded. | The recruiting and treating doctors, as well as the patients, were not blinded on the type of treatment throughout the medication period. | No information was provided. | The trial did not report any dropouts. | Results of all primary outcomes were reported. | Unclear. |
| He L 2013 | The randomization  was performed through a preprinted randomization table. | No information was provided. | No information was provided. | No information was provided. | The trial did not report any dropouts. | Results of all primary outcomes were reported. | Unclear. |
| Xu J 2013 | The trial only reputed randomized”. | No information was provided. | No information was provided. | No information was provided. | The trial did not report any dropouts. | Results of all primary outcomes were reported. | Unclear. |
| Li M 2015 | The trial only reported randomized” | No information was provided. | No information was provided. | No information was provided. | The trial did not report any dropouts. | Results of all primary outcomes were reported. | Unclear. |
| Ramachandran 2016 | Participants were randomly assigned following computer-based random numbers to one of the two treatment groups. | The author who performed the randomization did not participate in the enrolment and allocation of treatment to the participants and concealment was done using sequentially labeled sealed envelopes containing the specified intervention. | Both the patients, treating physicians, and individuals were not blinded to patient treatment assignments. | Both the patients, treating physicians, and individuals assessing clinical outcomes and analyzing data. | The trial did not report any dropouts. | Results of all primary outcomes were reported. | Unclear. |
| Omrani 2017 | The trial only reputed randomized”. | No information was provided. | This article was a double-blind study. | No information was provided. | The trial did not report any dropouts. | Results of all primary outcomes were reported. | Unclear. |
| Liang Q 2017 | A few patients chose the regimen. | No information was provided. | No information was provided. | No information was provided. | The trial did not report any dropouts. | Results of all primary outcomes were reported. | Unclear. |
| Scolari 2021 | An analyst from a distant site, with no clinical involvement in the trial, generated the randomization lists. | An analyst from a distant site, with no clinical involvement in the trial, kept randomization lists concealed. | No information was provided. | No information was provided. | The trial did not report any dropouts. | Results of all primary outcomes were reported. | Unclear. |


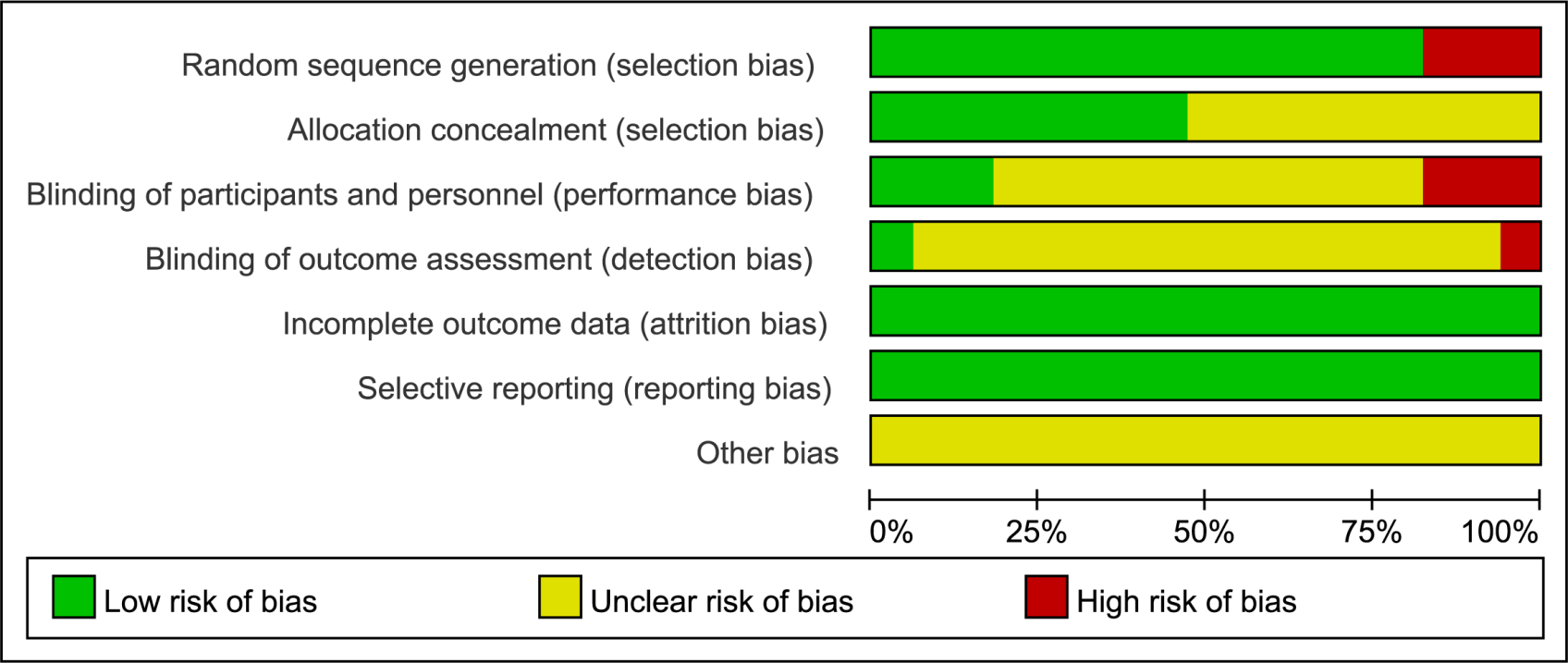


# eFig.1 Risk of bias graph

#
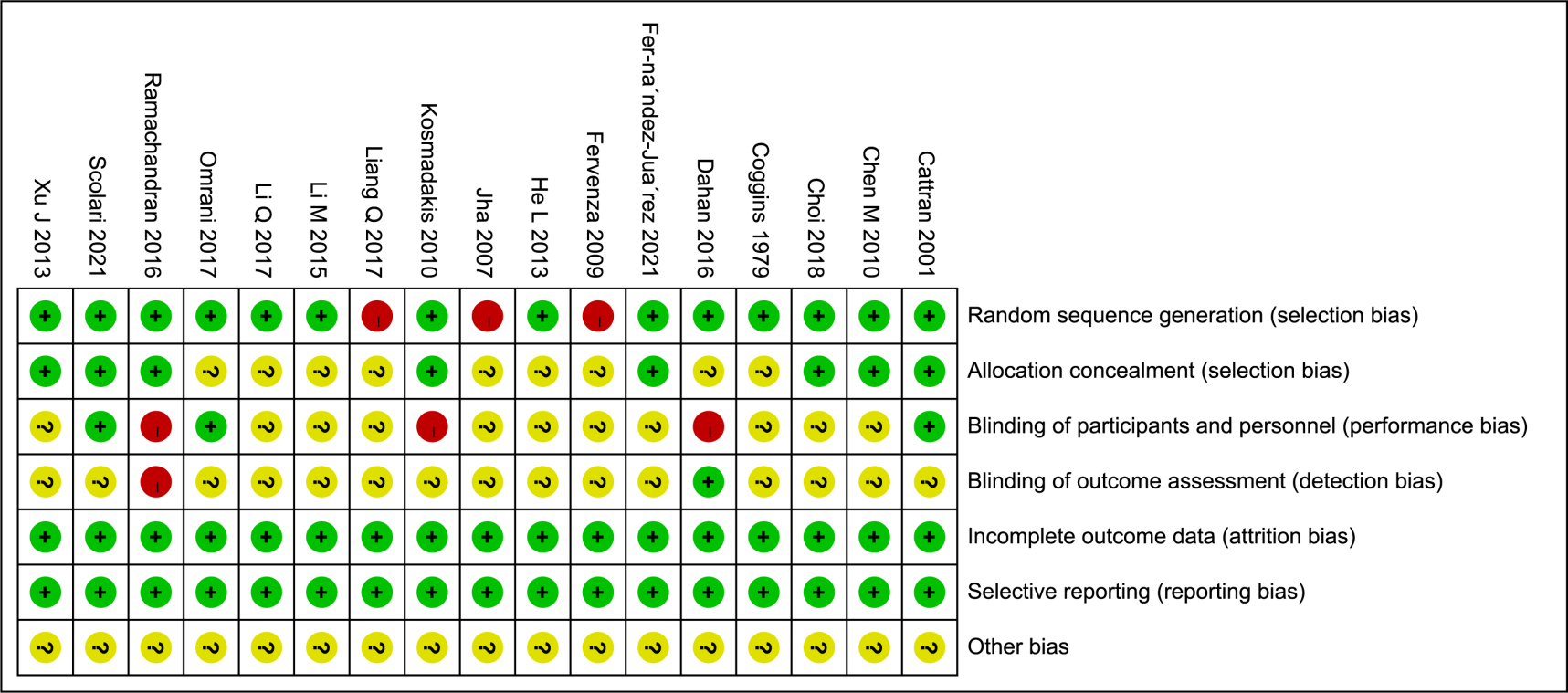


# eFig.2 Risk of bias summary

eTable2. Quality evaluation of enrolled cohort studies according to Newcastle-Ottawa Scale (NOS)

| **Study** | **Selection** | | | | **Comparability** | **Outcomes** | | | **Scores** |
| --- | --- | --- | --- | --- | --- | --- | --- | --- | --- |
|  | representativeness of the exposed cohort | Selection of unexposed cohort | ascertainment of exposure | Demonstration that outcome of interest was not present at the start of the study | Comparability of cohort-based on design or analysis | Assessment of outcome | Was follow-up long enough for the disease studied? | Adequacy of follow up of cohorts |  |
| Nayagam 2008 | ★ | - | ★ | ★ | ★ | ★ | ★ | - | 6 |
| Alexopoulos 2006 | ★ | ★ | ★ | ★ | ★ | - | ★ | - | 6 |
| Shin 2012 | ★ | ★ | ★ | ★ | ★ | ★ | ★ | ★ | 8 |
| Medrano 2015 | ★ | - | ★ | ★ | ★ | ★ | ★ | - | 6 |
| Peng L 2015 | ★ | ★ | ★ | ★ | ★★ | ★ | ★ | - | 8 |
| van den Brand 2017 | ★ | - | ★ | ★ | ★ | ★ | ★ | ★ | 7 |
| Li C 2018 | ★ | ★ | ★ | ★ | ★ | ★ | ★ | ★ | 8 |

# A maximum of 2 stars can be allotted in this category, and starts represent two different types of intervention.

eTable3. Quality evaluation of enrolled case-control study according to Newcastle-Ottawa Scale (NOS)

| **Study** | **Selection** | | | | **Comparability** | **Exposure** | | | **Scores** |
| --- | --- | --- | --- | --- | --- | --- | --- | --- | --- |
|  | An adequate definition of the cases? | Representativeness of the cases | Selection of controls | Definition of controls | Control for an important factor | Ascertainment of exposure | The same method of ascertainment for cases and controls | Non-Response rate |  |
| Fenoglio 2021 | ★ | ★ | - | ★ | ★ | - | ★ | ★ | 6 |

A maximum of 2 stars can be allotted in this category, and starts represent two different types of intervention.
