# Supplementary material for: Treatment of Idiopathic Membranous Nephropathy for Moderate or Severe Proteinuria: A Systematic Review and Network Meta-Analysis
Source: Int J Clin Pract. 2022 Apr 23;2022:4996239. doi: 10.1155/2022/4996239 (PMC9159126; doi:10.1155/2022/4996239)
Supplement: Supplementary Materials — Supplement 1. The selection criteria with a “PICOS” structure for the enrolled studies. Supplement 2. Risk of bias table for included studies. Supplement 3. Evaluation of inconsistency for outcomes. Supplement 4. Evaluation of heterogeneity analysis. Supplement 5. Results from pairwise meta-analyses. Supplement 6. The occurrence of adverse events. Supplement 7. Evaluation of meta-regression. Supplement 8. Net-funnel of publication bias. [file 4996239.f1.zip › 4996239.f1/Supplement 4 (1).docx]

***Supplementary material 4: Evaluation of heterogeneity analysis.***

1. **Total remission（pre-study proteinuria>8g/d）**

**
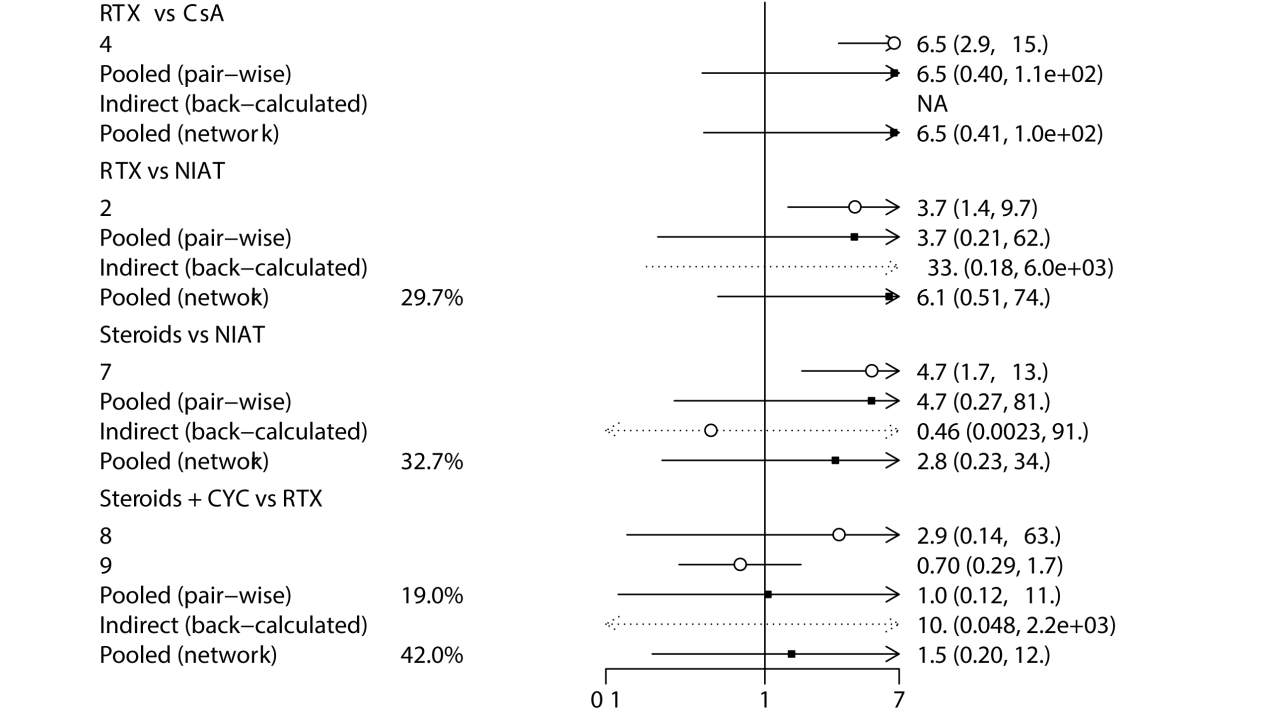
**

**
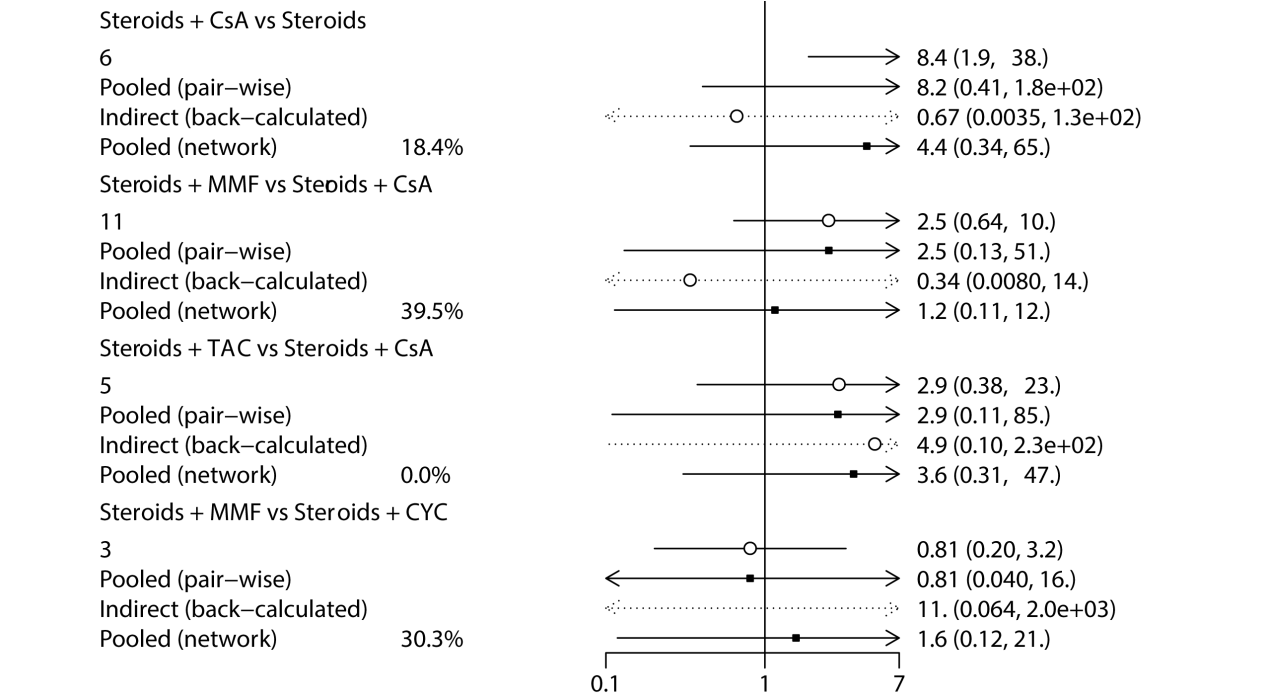
**

**
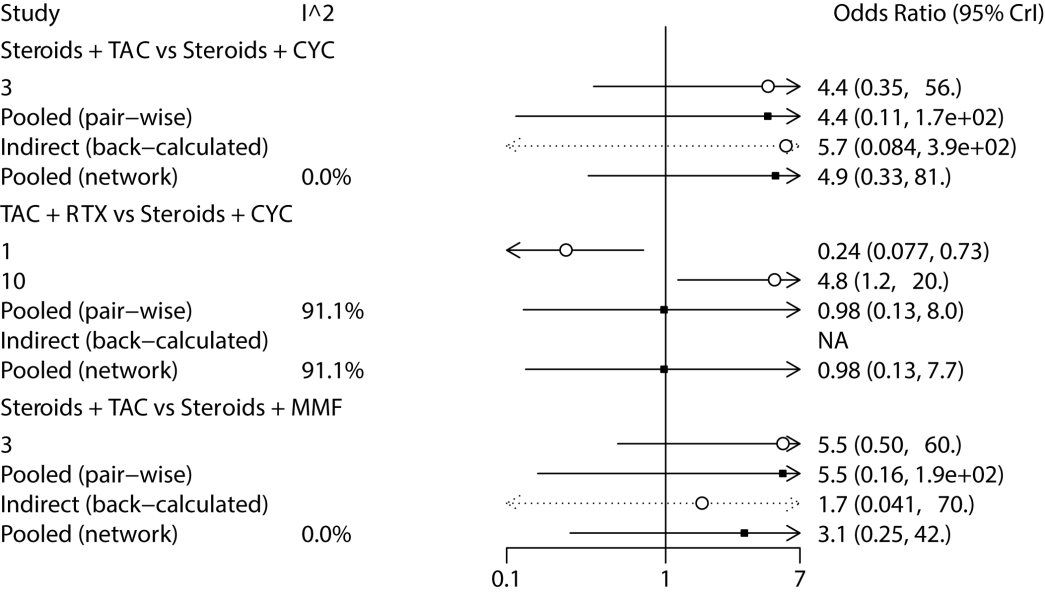
**

1. **Total remission（pre-study proteinuria < 8g/d）**

**
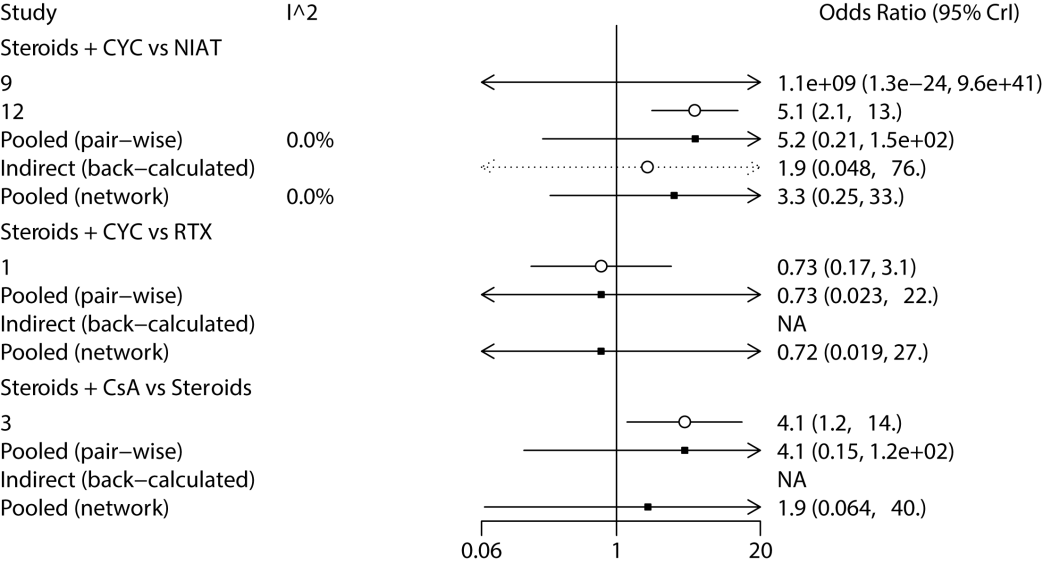
**


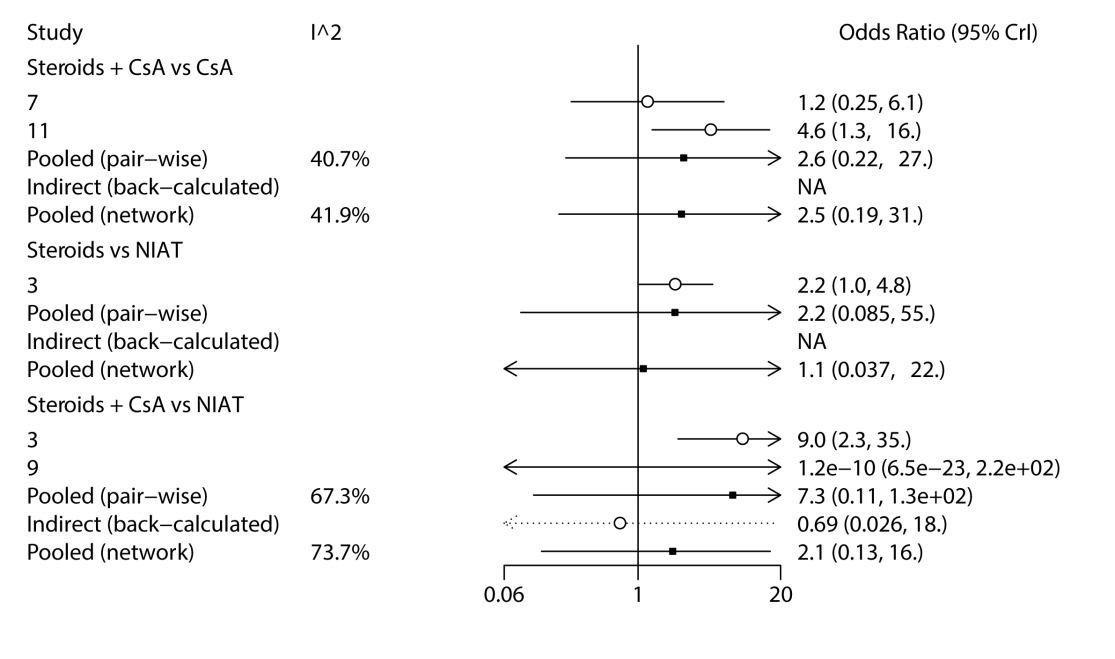

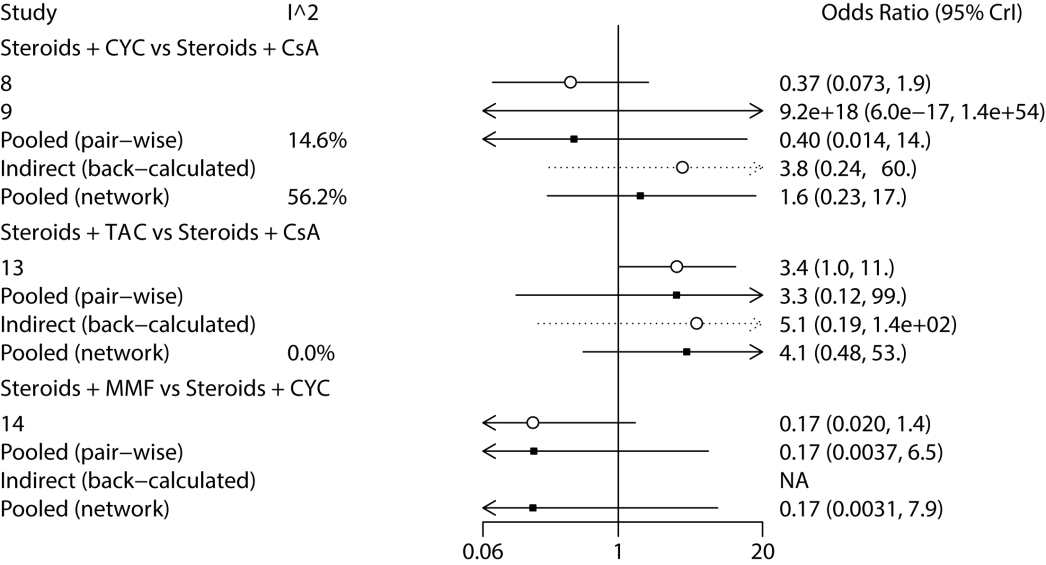


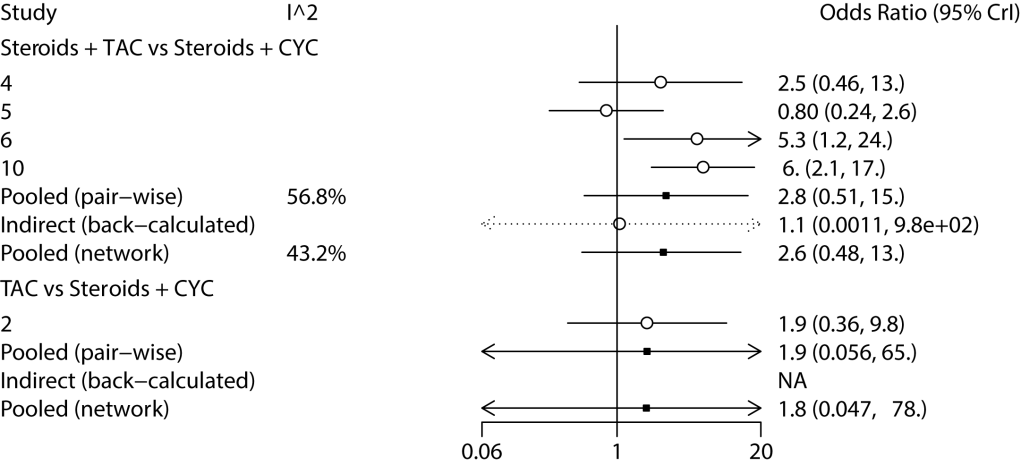


1. **Bone marrow suppression**

**
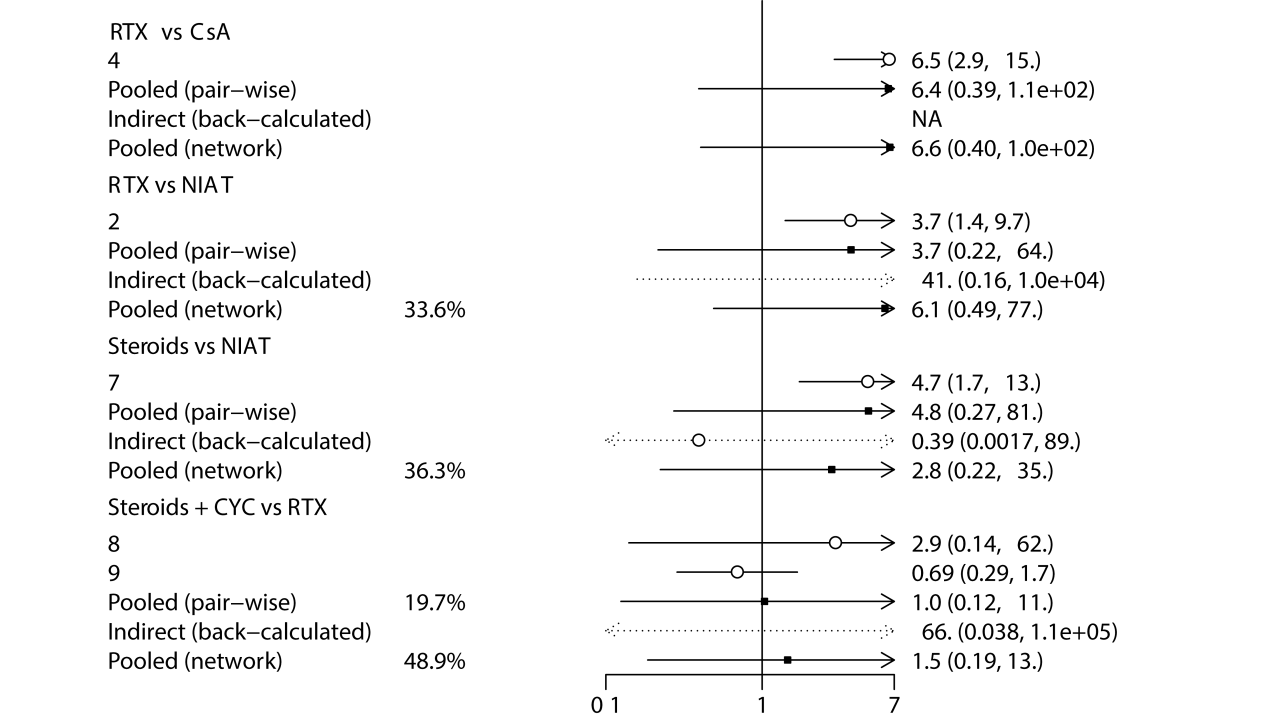
**

**
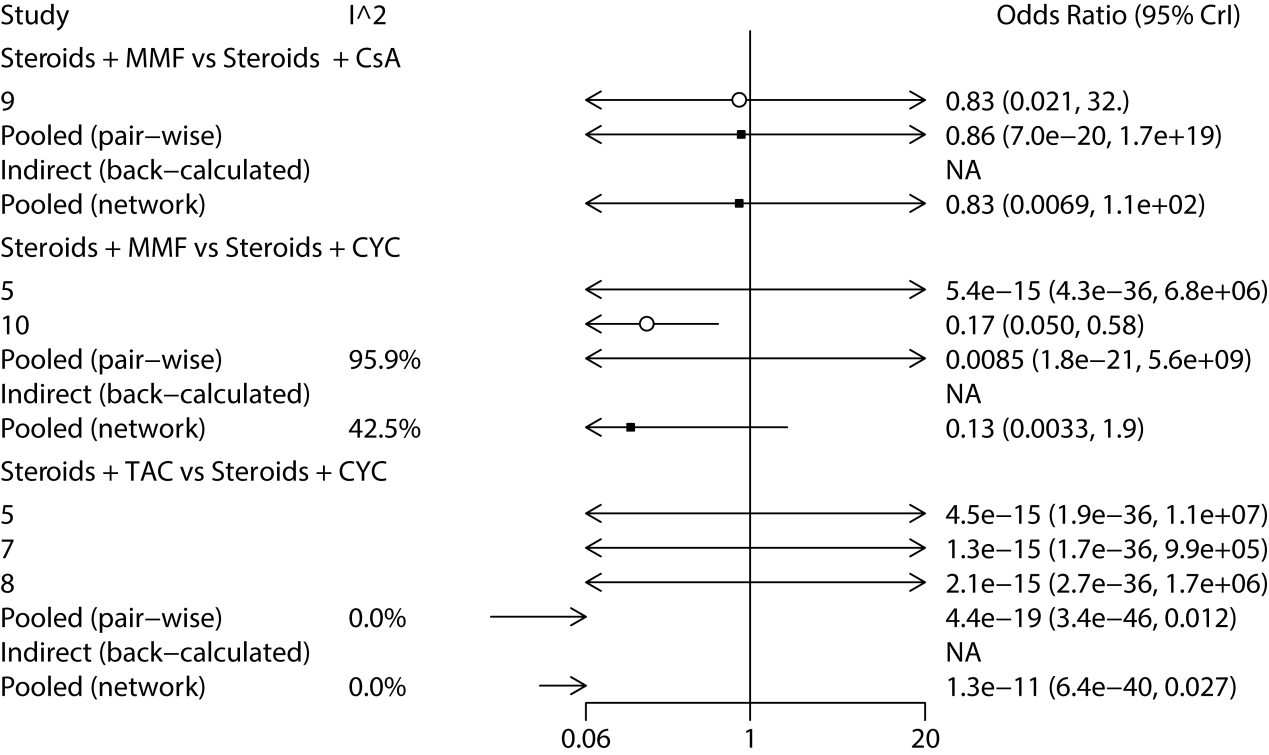
**

**
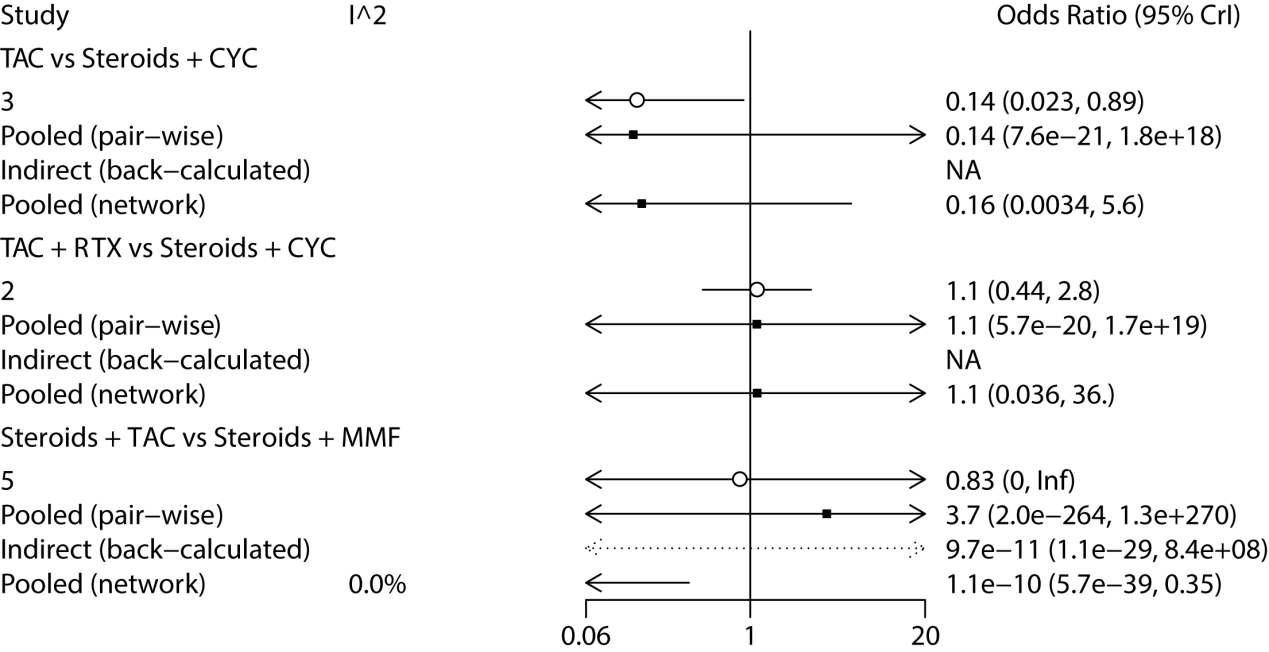
**

1. **Gastrointestinal symptoms**

**
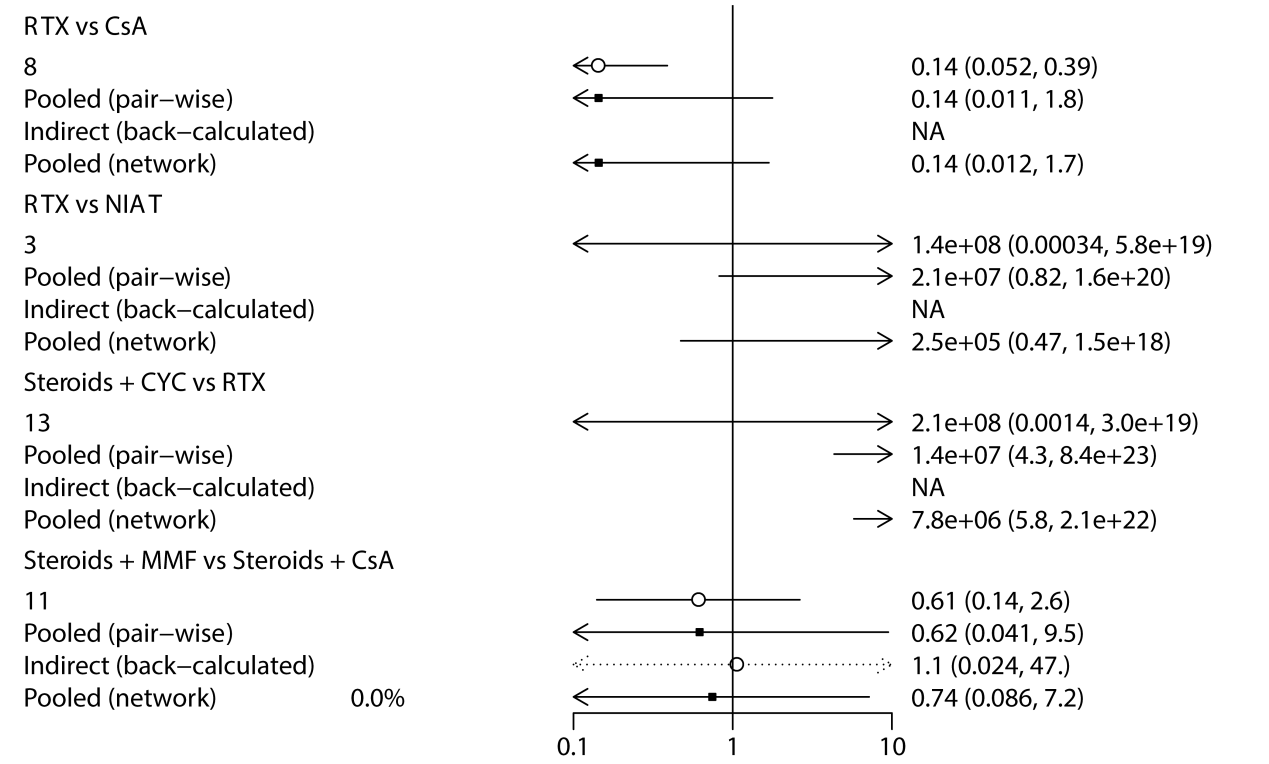
**

**
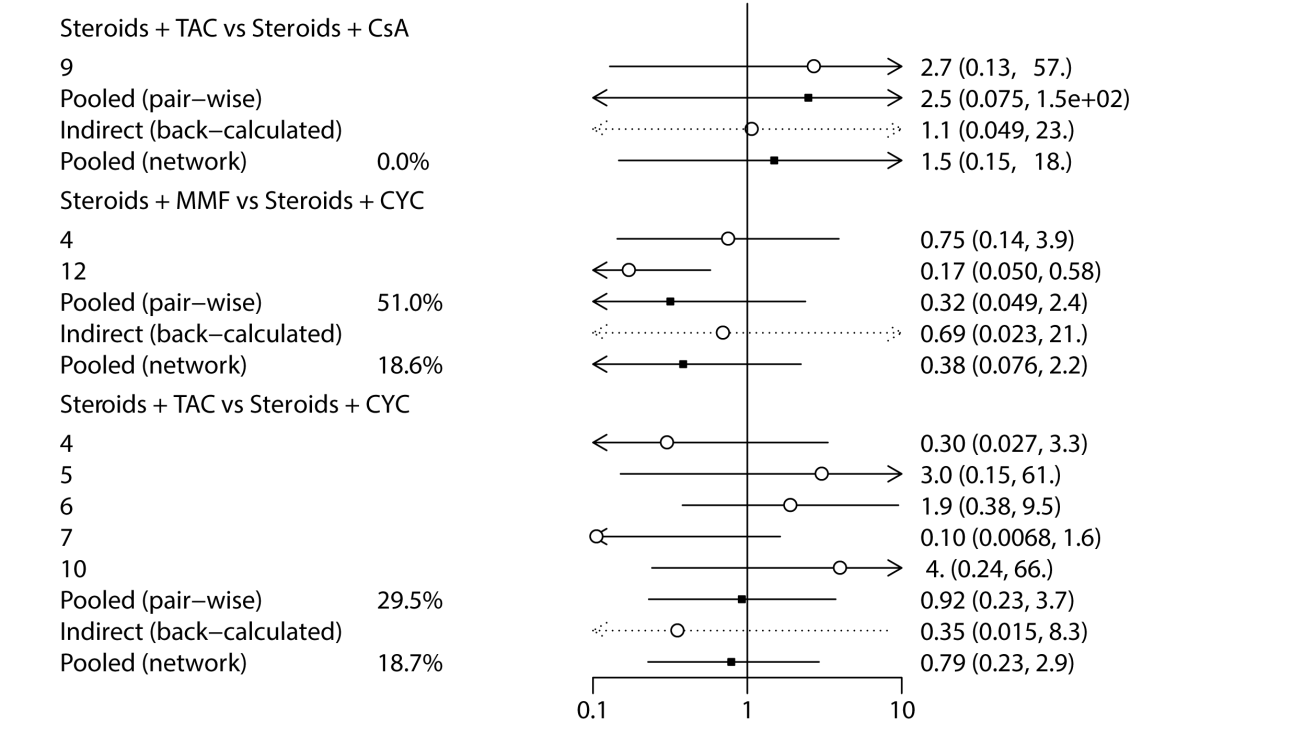
**

**
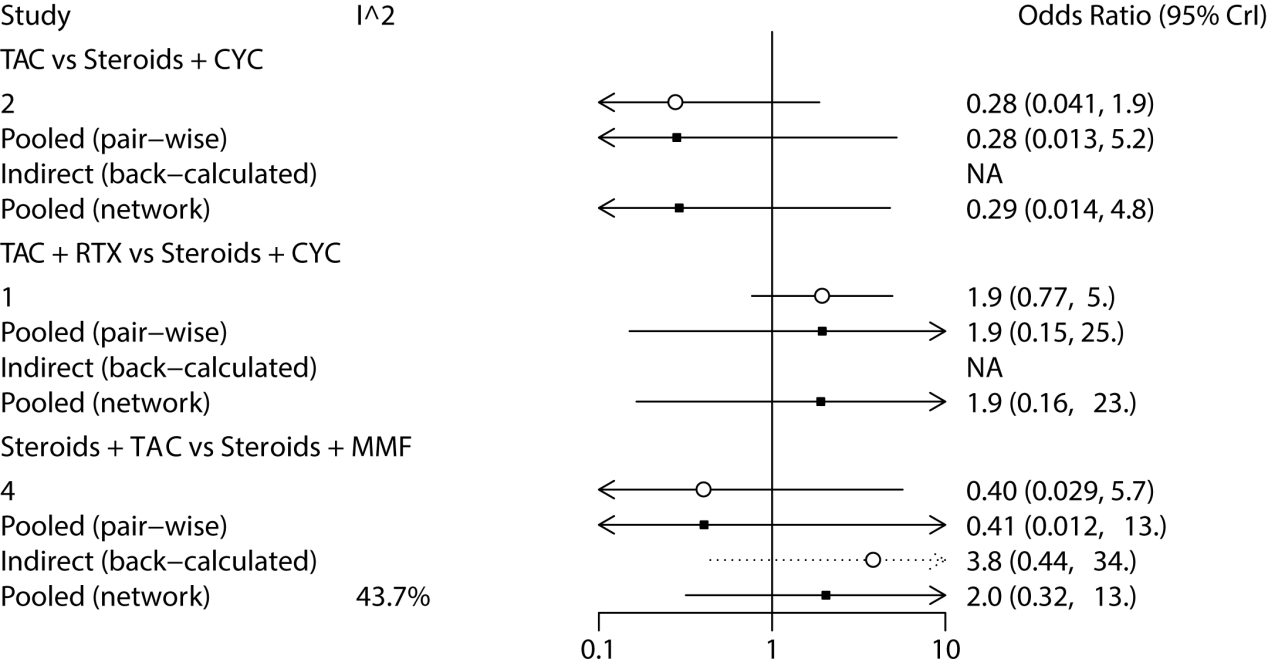
**
