# Supplementary material for: Treatment of Idiopathic Membranous Nephropathy for Moderate or Severe Proteinuria: A Systematic Review and Network Meta-Analysis
Source: Int J Clin Pract. 2022 Apr 23;2022:4996239. doi: 10.1155/2022/4996239 (PMC9159126; doi:10.1155/2022/4996239)
Supplement: Supplementary Materials — Supplement 1. The selection criteria with a “PICOS” structure for the enrolled studies. Supplement 2. Risk of bias table for included studies. Supplement 3. Evaluation of inconsistency for outcomes. Supplement 4. Evaluation of heterogeneity analysis. Supplement 5. Results from pairwise meta-analyses. Supplement 6. The occurrence of adverse events. Supplement 7. Evaluation of meta-regression. Supplement 8. Net-funnel of publication bias. [file 4996239.f1.zip › 4996239.f1/Supplement 5.docx]

***Supplement 5. Results from pairwise meta-analyses***

eTable 1 Pairwise meta-analysis results of total remission (pre-study proteinuria > 8g/d)

| **Contrast** | **No. of study** | **OR** | **95%CI** | **Q** | **tau^2^** | **I^2^** |
| --- | --- | --- | --- | --- | --- | --- |
| Steroids + CsA vs Steroids + MMF | 1 | 1.333 | (0.509, 3.490) | 0 | - | - |
| Steroids + CYC vs TAC + RTX | 2 | 0.935 | (0.521, 1.678) | 1.39 | 0.0502 | 28.2% |
| Steroids + CYC vs RTX | 2 | 1.026 | (0.704, 1.496) | 0.04 | 0 | 0.0% |
| NIAT vs Steroids | 1 | 2.235 | (0.947, 5.277) | 0 | - | - |
| Steroids + CsA vs Steroids | 1 | 0.231 | (0.059, 0.899) | 0 | - | - |
| Steroids + TAC vs Steroids + CsA | 1 | 0.838 | (0.291, 2.415) | 0 | - | - |
| CsA vs RTX | 1 | 3.000 | (1.463, 6.153) | 0 | - | - |
| Steroids + CYC vs Steroids + TAC | 1 | 1.185 | (0.551, 2.549) | 0 | - | - |
| Steroids + CYC vs Steroids + MMF | 1 | 0.955 | (0.431, 2.114) | 0 | - | - |
| Steroids + TAC vs Steroids + MMF | 1 | 0.806 | (0.373, 1.742) | 0 | - | - |
| NIAT vs RTX | 1 | 1.896 | (0.841, 4.274) | 0 | - | - |

Note: RTX, rituximab; CsA, [cyclosporin](javascript:;) [A;](javascript:;) CYC, cyclophosphamide; TAC, tacrolimus; NIAT, 6 months of nonimmunosuppressive antiproteinuric treatment; MMF, [Mycophenolate Mofetil](javascript:;).

eTable 1 Pairwise meta-analysis results of total remission (pre-study proteinuria < 8g/d)

| **Contrast** | **No. of study** | **OR** | **95%CI** | **Q** | **tau^2^** | **I^2^** |
| --- | --- | --- | --- | --- | --- | --- |
| Steroids + CYC vs RTX | 1 | 1.049 | (0.484, 2.276) | 0 | - | - |
| Steroids + CYC vs TAC | 1 | 1.082 | (0.497, 2.358) | 0 | - | - |
| Steroids + CsA vs NIAT | 2 | 0.779 | (0.243, 2.495) | 2.64 | 0.4657 | 62.1% |
| Steroids + CsA vs Steroids | 1 | 0.711 | (0.408, 1.236) | 0 | - | - |
| NIAT vs Steroids | 1 | 1.451 | (0.791, 2.662) | 0 | - | - |
| Steroids + CYC vs Steroids + TAC | 4 | 1.269 | (0.872, 1.846) | 2.15 | 0 | 0.0% |
| Steroids + CsA vs CsA | 2 | 0.724 | (0.372, 1.407) | 0.22 | 0 | 0.0% |
| Steroids + CYC vs Steroids + CsA | 3 | 0.985 | (0.473, 2.050) | 0.65 | 0 | 0.0% |
| Steroids + CYC vs NIAT | 2 | 0.569 | (0.303, 1.070) | 0.92 | 0 | 0.0% |
| Steroids + TAC vs Steroids + MMF | 1 | 0.759 | (0.366, 1.574) | 0 | - | - |
| Steroids + CYC vs Steroids + MMF | 1 | 0.568 | (0.139, 2.322) | 0 | - | - |

Note: RTX, rituximab; CsA, [cyclosporin](javascript:;) [A;](javascript:;) CYC, cyclophosphamide; TAC, tacrolimus; NIAT, 6 months of nonimmunosuppressive antiproteinuric treatment; MMF, [Mycophenolate Mofetil](javascript:;).

eTable 1 Pairwise meta-analysis results of bone marrow suppression

| **Contrast** | **No. of study** | **OR** | **95%CI** | **Q** | **tau^2^** | **I^2^** |
| --- | --- | --- | --- | --- | --- | --- |
| Steroids + CYC vs RTX | 2 | 0.073 | (0.009, 0.562) | 0 | 0 | 0.0% |
| Steroids + CYC vs TAC + RTX | 1 | 1.077 | (0.453, 2.558) | 0 | - | - |
| Steroids + CYC vs TAC | 1 | 0.233 | (0.046, 1.194) | 0 | - | - |
| Steroids + CYC vs Steroids + TAC | 3 | 0.156 | (0.027, 0.908) | 0.34 | 0 | 0.0% |
| Steroids + CYCvsSteroids + MMF | 2 | 0.315 | (0.109, 0.905) | 0 | 0 | 0.0% |
| Steroids + CsA vs NIAT | 1 | 0.176 | (0.008, 3.745) | 0 | - | - |
| Steroids + CsA vs Steroids | 1 | 0.139 | (0.007, 2.964) | 0 | - | - |
| Steroids + CsA vs Steroids + MMF | 1 | 0.857 | (0.050 , 14.706) | 0 | - | - |

Note: RTX, rituximab; CsA, [cyclosporin](javascript:;) [A;](javascript:;) CYC, cyclophosphamide; TAC, tacrolimus; NIAT, 6 months of nonimmunosuppressive antiproteinuric treatment; MMF, [Mycophenolate Mofetil](javascript:;).

eTable 1 Pairwise meta-analysis results of gastrointestinal symptoms

| **Contrast** | **No. of study** | **OR** | **95%CI** | **Q** | **tau^2^** | **I^2^** |
| --- | --- | --- | --- | --- | --- | --- |
| Steroids + CYC vs TAC + RTX | 1 | 1.545 | (0.649, 3.682) | 0 | - | - |
| Steroids + CYC vs TAC | 1 | 0.373 | (0.067, 2.082) | 0 | - | - |
| NIAT vs RTX | 1 | 3.080 | (0.122, 78.021) | 0 | - | - |
| Steroids + CYC vs Steroids + TAC | 5 | 1.248 | (0.353, 4.417) | 8.53 | 1.0896 | 53.1% |
| Steroids + CYC vs Steroids + MMF | 2 | 0.452 | (0.184, 1.110) | 1.05 | 0.0210 | 4.7% |
| Steroids + TAC vs Steroids + MMF | 1 | 2.000 | (0.340, 11.756) | 0 | - | - |
| CsA vs RTX | 1 | 0.231 | (0.089, 0.598) | 0 | - | - |
| Steroids + TAC vs Steroids + CsA | 1 | 0.533 | (0.044, 6.508) | 0 | - | - |
| Steroids + CsA vs Steroids + MMF | 1 | 0.714 | (0.186, 2.737) | 0 | - | - |
| Steroids + CYC vs RTX | 1 | 0.114 | (0.006, 2.153) | 0 | - | - |

Note: RTX, rituximab; CsA, [cyclosporin](javascript:;) [A;](javascript:;) CYC, cyclophosphamide; TAC, tacrolimus; NIAT, 6 months of nonimmunosuppressive antiproteinuric treatment; MMF, [Mycophenolate Mofetil](javascript:;).
