# Supplementary material for: Treatment of Idiopathic Membranous Nephropathy for Moderate or Severe Proteinuria: A Systematic Review and Network Meta-Analysis
Source: Int J Clin Pract. 2022 Apr 23;2022:4996239. doi: 10.1155/2022/4996239 (PMC9159126; doi:10.1155/2022/4996239)
Supplement: Supplementary Materials — Supplement 1. The selection criteria with a “PICOS” structure for the enrolled studies. Supplement 2. Risk of bias table for included studies. Supplement 3. Evaluation of inconsistency for outcomes. Supplement 4. Evaluation of heterogeneity analysis. Supplement 5. Results from pairwise meta-analyses. Supplement 6. The occurrence of adverse events. Supplement 7. Evaluation of meta-regression. Supplement 8. Net-funnel of publication bias. [file 4996239.f1.zip › 4996239.f1/Supplement 7.docx]

***Supplementary material 7: Evaluation of meta-regression***

eTable1. Results of meta-regression for TR (proteinuria > 8g/d)

| Variables | Comparison | Regression coefficient | CIs |
| --- | --- | --- | --- |
| Age | NIAT vs RTX | 0.43 | (-1.76, 1.82) |
|  | NIAT vs Steroids | -0.55 | (-1.67, 0.73) |
|  | RTX vs CsA | -1.72 | (-3.11, 0.46) |
|  | RTX vs Steroids + CYC | -0.31 | (-1.53, 0.94) |
|  | Steroids vs Steroids + CsA | 0.17 | (-0.87, 1.19) |
|  | Steroids + CYC vs Steroids + MMF | 1.22 | (-0.32, 2.44) |
|  | Steroids + CYC vs Steroids + TAC | 0.67 | (-1.67, 1.90) |
|  | Steroids + CYC vs TAC + RTX | -3.78 | (-6.66, -0.27) |
| Study duration | NIAT vs RTX | 0.19222 | (-1.82, 1.77) |
|  | NIAT vs Steroids | -0.39451 | (-1.56, 0.83) |
|  | RTX vs CsA | -1.83192 | (-3.34, 0.45) |
|  | RTX vs Steroids + CYC | -0.21279 | (-1.45, 0.99) |
|  | Steroids vs Steroids + CsA | 0.33657 | (-0.77, 1.31) |
|  | Steroids + CYC vs Steroids + MMF | 1.39136 | (-0.25, 2.67) |
|  | Steroids + CYC vs Steroids + TAC | -0.09768 | (-1.81, 1.69) |
|  | Steroids + CYC vs TAC + RTX | -3.69515 | (-6.38, -0.29) |

[Abbreviation](javascript:;): RTX, rituximab; CsA, [cyclosporin](javascript:;) [A;](javascript:;) CYC, cyclophosphamide; TAC, tacrolimus; NIAT, nonimmunosuppressive antiproteinuric treatment; MMF, [mycophenolate mofetil](javascript:;).

eTable2. Results of meta-regression for TR (proteinuria < 8g/d)

| Variables | Comparison | Regression coefficient | CIs |
| --- | --- | --- | --- |
| Age | Steroids + CsA vs CsA | -3.235e-01 | (-1.77,1.23) |
|  | Steroids + CsA vs NIAT | -8.995e-01 | (-2.89,2.56) |
|  | Steroids + CsA vs Steroids | 2.534e-01 | (-2.28,2.43) |
|  | Steroids + CsA vs Steroids + CYC | 1.176e+00 | (-0.10,2.36) |
|  | Steroids + CsA vs Steroids + TAC | 1.822e+00 | (0.45,3.02) |
|  | Steroids + CYC vs RTX | -2.244e+00 | (-2.83,2.31) |
|  | Steroids + CYC vs Steroids + MMF | 1.280e+00 | (-0.14,2.54) |
|  | Steroids + CYC vs TAC | -1.331e+00 | (-2.78,2.44) |
| Study duration | Steroids + CsA vs CsA | -0.3003 | (-1.71, 1.21) |
|  | Steroids + CsA vs NIAT | -0.0354 | (-2.54, 2.50) |
|  | Steroids + CsA vs Steroids | -10.0265 | (-3.12, 2.42) |
|  | Steroids + CsA vs Steroids + CYC | -2.0275 | (-3.37,-0.46) |
|  | Steroids + CsA vs Steroids + TAC | 0.4780 | (-0.88, 1.69) |
|  | Steroids + CYC vs RTX | 1.3932 | (-2.15, 2.81) |
|  | Steroids + CYC vs Steroids + MMF | 0.8262 | (-0.65, 2.16) |
|  | Steroids + CYC vs TAC | -0.2936 | (-2.66, 2.60) |

[Abbreviation](javascript:;): RTX, rituximab; CsA, [cyclosporin](javascript:;) [A;](javascript:;) CYC, cyclophosphamide; TAC, tacrolimus; NIAT, nonimmunosuppressive antiproteinuric treatment; MMF, [mycophenolate mofetil](javascript:;).

eTable3. Results of meta-regression for bone marrow suppression

| Variables | Comparison | Regression coefficient | CIs |
| --- | --- | --- | --- |
| Age | Steroids + CsA vs NIAT | 0.05 | (-2.57, 2.49) |
|  | Steroids + CsA vs Steroids | -1.35 | (-2.90, 2.56) |
|  | Steroids + CYC vs RTX | 0.70 | (-2.40, 2.98) |
|  | Steroids + CYC vs TAC | -2.50 | (-4.64, 0.63) |
|  | Steroids + CYC vs TAC + RTX | 6.30 | (-0.34, 5.25) |
|  | Steroids + MMF vs Steroids + CsA | 3.61 | (-2.18, 3.23) |
|  | Steroids + MMF vs Steroids + CYC | -0.67 | (-2.65, 2.69) |
|  | Steroids + MMF vs Steroids + TAC | -1.07 | (-2.80, 2.14) |
| Study duration | Steroids + CsA vs NIAT | -0.24 | (-2.53, 2.46) |
|  | Steroids + CsA vs Steroids | -31.81 | (-4.43, 1.95) |
|  | Steroids + CYC vs RTX | 4.18 | (-2.00,4.02) |
|  | Steroids + CYC vs TAC | -2.46 | (-3.77, 1.00) |
|  | Steroids + CYC vs TAC + RTX | 3.04 | (-0.78, 4.15) |
|  | Steroids + MMF vs Steroids + CsA | 6.05 | (-2.16, 3.26) |
|  | Steroids + MMF vs Steroids + CYC | 0.73 | (-2.57, 2.79) |
|  | Steroids + MMF vs Steroids + TAC | 0.29 | (-2.47, 2.53) |
| Pre-study proteinuria | Steroids + CsA vs NIAT | 0.78 | (-2.18, 2.68) |
|  | Steroids + CsA vs Steroids | 9.41 | (-2.26, 3.40) |
|  | Steroids + CYC vs RTX | -0.24 | (-2.76, 2.30) |
|  | Steroids + CYC vs TAC | 1.79 | (-0.63, 3.49) |
|  | Steroids + CYC vs TAC + RTX | -3.71 | (-4.58, 0.47) |
|  | Steroids + MMF vs Steroids + CsA | -0.08 | (-2.47, 2.57) |
|  | Steroids + MMF vs Steroids + CYC | -0.14 | (-2.87, 2.72) |
|  | Steroids + MMF vs Steroids + TAC | 0.81 | (-2.38, 3.06) |

[Abbreviation](javascript:;): RTX, rituximab; CsA, [cyclosporin](javascript:;) [A;](javascript:;) CYC, cyclophosphamide; TAC, tacrolimus; NIAT, nonimmunosuppressive antiproteinuric treatment; MMF, [mycophenolate mofetil](javascript:;).

eTable4. Results of meta-regression for gastrointestinal symptoms

| Variables | Comparison | Regression coefficient | CIs |
| --- | --- | --- | --- |
| Age | RTX vs CsA | 1.03372 | (-2.18, 2.26) |
|  | RTX vs NIAT | -0.65998 | (-2.48, 1.98) |
|  | Steroids + CYC vs RTX | 0.18681 | (-0.84, 1.20) |
|  | Steroids + CYC vs Steroids + MMF | 0.17820 | (-0.70, 1.04) |
|  | Steroids + CYC vs Steroids + TAC | -0.99174 | (-1.93, 0.00) |
|  | Steroids + CYC vs TAC | 0.68644 | (-0.31, 1.63) |
|  | Steroids + CYC vs TAC + RTX | 0.50974 | (-2.12, 2.23) |
|  | Steroids + MMF vs Steroids + CsA | -6.01866 | (-2.81, 1.75) |
| Study duration | RTX vs CsA | -1.37 | (-2.36, 1.84) |
|  | RTX vs NIAT | -0.21 | (-2.15, 1.75) |
|  | Steroids + CYC vs RTX | 0.70 | (-0.67, 1.96) |
|  | Steroids + CYC vs Steroids + MMF | -0.24 | (-1.15, 0.71) |
|  | Steroids + CYC vs Steroids + TAC | -1.14 | (-2.09, -0.08) |
|  | Steroids + CYC vs TAC | 0.88 | (-0.14, 1.88) |
|  | Steroids + CYC vs TAC + RTX | 0.97 | (-2.10, 2.26) |
|  | Steroids + MMF vs Steroids + CsA | -0.27 | (-2.24, 1.88) |
| Pre-study proteinuria | RTX vs CsA | -0.73156 | (-2.23, 2.00) |
|  | RTX vs NIAT | -0.69065 | (-2.02, 1.99) |
|  | Steroids + CYC vs RTX | -0.60304 | (-2.04, 2.01) |
|  | Steroids + CYC vs Steroids + MMF | 0.02596 | (-0.94, 1.01) |
|  | Steroids + CYC vs Steroids + TAC | -0.91898 | (-1.96, 0.20) |
|  | Steroids + CYC vs TAC | 0.77411 | (-0.28, 1.78) |
|  | Steroids + CYC vs TAC + RTX | 0.60212 | (-2.13, 2.10) |
|  | Steroids + MMF vs Steroids + CsA | 0.25946 | (-2.03, 2.06) |

[Abbreviation](javascript:;): RTX, rituximab; CsA, [cyclosporin](javascript:;) [A;](javascript:;) CYC, cyclophosphamide; TAC, tacrolimus; NIAT, nonimmunosuppressive antiproteinuric treatment; MMF, [mycophenolate mofetil](javascript:;).
